# Laboratory and microcosm experiments reveal contrasted adaptive responses to ammonia and water mineralisation in aquatic stages of the sibling species *Anopheles gambiae* (sensu stricto) and *Anopheles coluzzii*

**DOI:** 10.1186/s13071-020-04483-7

**Published:** 2021-01-06

**Authors:** Nwamaka Oluchukwu Akpodiete, Frédéric Tripet

**Affiliations:** Centre for Applied Entomology and Parasitology, School of Life Sciences, Keele University, Staffordshire, UK

**Keywords:** G × E interactions, Eco-speciation, Malaria, Rice fields, *Anopheles gambiae*, *Anopheles coluzzii*, Ammonia tolerance, Mineral water

## Abstract

**Background:**

The sibling species of the malaria mosquito, *Anopheles gambiae* (sensu stricto) and *Anopheles coluzzii* co-exist in many parts of West Africa and are thought to have recently diverged through a process of ecological speciation with gene flow. Divergent larval ecological adaptations, resulting in Genotype-by-Environment (G × E) interactions, have been proposed as important drivers of speciation in these species. In West Africa, *An. coluzzii* tends to be associated with permanent man-made larval habitats such as irrigated rice fields, which are typically more eutrophic and mineral and ammonia-rich than the temporary rain pools exploited by *An. gambiae* (s.s.)

**Methods:**

To highlight G × E interactions at the larval stage and their possible role in ecological speciation of these species, we first investigated the effect of exposure to ammonium hydroxide and water mineralisation on larval developmental success. Mosquito larvae were exposed to two water sources and increasing ammonia concentrations in small containers until adult emergence. In a second experiment, larval developmental success was compared across two contrasted microcosms to highlight G × E interactions under conditions such as those found in the natural environment.

**Results:**

The first experiment revealed significant G × E interactions in developmental success and phenotypic quality for both species in response to increasing ammonia concentrations and water mineralisation. The *An. coluzzii* strain outperformed the *An. gambiae* (s.s.) strain under limited conditions that were closer to more eutrophic habitats. The second experiment revealed divergent crisscrossing reaction norms in the developmental success of the sibling species in the two contrasted larval environments. As expected, *An. coluzzii* had higher emergence rates in the rice paddy environment with emerging adults of superior phenotypic quality compared to *An. gambiae* (s.s.), and vice versa, in the rain puddle environment.

**Conclusions:**

Evidence for such G × E interactions lends support to the hypothesis that divergent larval adaptations to the environmental conditions found in man-made habitats such as rice fields in *An. coluzzii* may have been an important driver of its ecological speciation. 
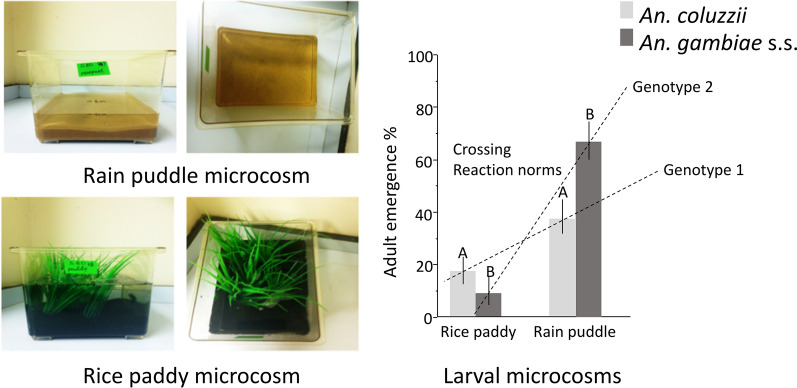

## Background

Sub-Saharan Africa is malaria-endemic, with 93% worldwide incidence and 85% of malaria mortality occurring in this region in 2018 [1]. The recently speciated sibling species, *Anopheles gambiae* (sensu stricto) and *Anopheles coluzzii*, members of the *An. gambiae* (sensu lato) complex, are the major vectors of malaria in this region [1]. They are very efficient vectors of malaria because of their close association with human dwellings, with larval habitats resulting from anthropogenic sources as well as their endophilic and endophagic behaviour [2]. Both species are morphologically similar and share many behavioural and ecological traits, such as their vertebrate hosts’ preferences, exploitation of adult resting sites, and freshwater larval habitats. Although existing in sympatry, detailed studies have highlighted conspecific preferences in mating behaviour, resulting in strong assortative mating which reinforces reproductive isolation resulting in fewer hybrids and strengthening speciation with restricted gene flow [3, 4]. In addition to assortative mating, differences in oviposition site preference and larval adaptations, such as predator avoidance, pollution tolerance, and response to interspecific competition, have been highlighted as potential drivers of ecological speciation in these sibling species [5–8].

Ecological speciation occurs when a subset of individuals from a population evolves a new set of adaptive phenotypic responses to novel variable environments [9, 10]. When the original and derived populations interbreed, divergent adaptive responses can result in maladapted hybrids, selection for assortative mating, and restricted gene flow between them [11, 12]. Such differences in the genetic responses of two divergent species to their environment result in measurable Genotype-by-Environment (G × E) interactions or crisscrossing norms of reaction [9, 13].

In the sibling species, *An. gambiae* and *An. coluzzii*, one known ecological difference between the presumed ancestral *An. gambiae* (s.s.) and derived *An. coluzzii* is their larval habitat preferences. *Anopheles gambiae* (s.s.) principally exploits temporal rain-dependent larval habitats while *An. coluzzii* is often associated with man-made habitats such as irrigated rice fields [14]. This led to the hypothesis that the ancestral *An. coluzzii* might have been an ecotype adapted to more permanent breeding habitats. Subsequently, its process of divergence and expansion may have been closely associated with that of rice domestication by Neolithic West African populations 3000–3500 years ago [15–18]. Aquatic predators are more common in the *An. coluzzii* typical permanent habitats compared to the temporary shallow rain pools of *An. gambiae* (s.s.), which has prompted several studies focusing on G × E interactions of the two siblings species in response to predator presence [19, 20].

The preferred habitats of *An. gambiae* (s.s.) and *An. coluzzii* also differ in physicochemical composition with the more permanent larval habitats containing more minerals and organic matter as well as lower dissolved oxygen [21]. Due to high organic matter composition, permanent larval habitats such as rice fields have higher ammonia content [16, 22]. Ammonia is toxic to fish and aquatic invertebrates and is produced by prokaryotes either through diazotrophic fixation of atmospheric nitrogen and oxygen or as a by-product of protein metabolism and organic waste (ammonification) [23–25]. Ammonia is also produced by aquatic organisms through urea excretion and is subsequently converted to nitrite and less harmful nitrates through the process of nitrification by other microorganisms [25, 26]. Ammonia toxicity depends on its concentration as well as pH, temperature, and the level of water mineralisation which can buffer ammonia toxicity over pH 8 [23, 24].

The hypothesis that the larval stages of *An. coluzzii* and *An. gambiae* (s.s.) exhibit contrasting responses to ammonia has been investigated in an extensive survey of *An. gambiae* (s.l.) populations along gradients of urbanisation in Yaounde, Cameroon [27]. This study showed through acute toxicity bioassays that *An. coluzzii* has a higher ammonia tolerance than *An. gambiae* (s.s.), a difference which may explain the distribution of sibling species in aquatic habitats [27].

In this study, we investigated the adaptive responses of the aquatic stages of *An. coluzzii* and *An. gambiae* (s.s.) to ammonia and water mineralisation. First, we subjected immature stages of both species to increasing ammonia concentrations and two levels of mineralisation, until adult emergence. Second, we reared the sibling species in microcosms mimicking their preferred habitats, namely temporary rain puddles and rice field paddies, to highlight G × E interactions affecting developmental success and phenotypic quality. The crisscrossing reaction norms resulting from G × E interactions in the developmental success of the sibling species lend support to the hypothesis that adaptations to rice field-like conditions by *An. coluzzii* may have been an important driver of its ecological speciation.

## Methods

### Mosquito strains

Two strains were used for the experiments, the Kisumu strain of *An. gambiae* (s.s.), colonised over 40 years ago, from the area of Kisumu, Kenya, East Africa, and the 17-year-old Mopti strain of *An. coluzzii*, colonised in 2003 by the Lanzaro Laboratory (UC Davis) from the village of NʼGabacoro droit near Bamako, Mali, West Africa. Natural West African populations of the sibling species have recently introgressed with one another, resulting in the possible selective introgression of important pesticide resistance and ammonia detoxification loci [28, 29]. Therefore, in this study, we favoured older strains colonised before the intensification of chemical control with little evolutionary history of such recent anthropomorphic selection pressures and associated introgression [27, 29, 30].

### Strain maintenance

The strains were maintained by the Tripet group in dedicated insectaries at the Centre of Applied Entomology and Parasitology (CAEP), Keele University, UK. Mosquitoes were maintained at 25 ± 2 °C, a relative humidity of 70 ± 5%, with a 12 h light/dark photocycle. Larvae were fed an optimised diet of groundfish food (Tetramin; Tetra, Melle, Germany) at a rearing density of 200 larvae/litre by manual counting [31]. Pupae were transferred to 5l plastic tub cages (20.5 cm height × 20 cm diameter), covered with netting for adult emergence. Cages had sleeved opening for easy management of mosquitoes and accessories. Approximately 600–800 adults were held in a cage; sugar was provided via a paper towel soaked in 10% glucose solution and water via a soaked cotton pad in an upturned bowl placed on the cage netting. Female adult mosquitoes were fed with horse blood using an artificial feeding membrane (Hemotek feeding membrane system; Discovery workshops, Blackburn, UK). Styrofoam cups (egg cups) containing filter paper and water were placed in the cages 4 days after blood-feeding to collect eggs. Following the removal of the egg cups, the cages were washed thoroughly and sterilised with bleach. Mouth aspirators were used to transfer adults from one container to another when necessary.

### Experiment 1: effects of ammonia, feed regimes, and mineralisation on the developmental success of *An. gambiae* (s.s.) and *An. coluzzii*

#### Experimental design

The experimental design consisted of two species, two feeding regimes, two water types, and seven ammonia concentrations resulting in 2 × 2 × 2 × 7 treatment groups (Additional file 1: Figure S1). First-instar larvae of *An. coluzzii* and then *An. gambiae* (s.s.) were distributed using a 3 ml plastic pipette and kept in groups of ten larvae in white styrofoam cups containing 300 ml of water (at a 5 cm depth) with variable ammonia concentrations. Three replicates were made resulting in a total of 1680 (560 × 3) larvae used in the study. To keep the ammonia and mineralisation constant and unaffected by the accumulation of waste products, larvae from every group were transferred daily into new containers containing fresh water of their respective treatment group quality. At pupation, pupae were transferred to a netted styrofoam cup containing 100 ml of the same water quality but with no ammonia. The position of experimental pots was completely randomised and experimental cups labelled with codes to avoid bias due to variation in the insectary environment.

Two standard feeding regimes were used: solution or powder feed. On day 1, larvae from both feeding regime groups received a 0.1 μl single drop of Liquifry liquid fish food (Interpret Ltd., Surrey, UK). From day 2, half the cups were fed on a powder feeding regime that consisted of daily rations of TetraMin Baby powder fish food (Tetra GmbH, Melle, Germany). The rations increased over time and were dropped on the water surface using a micro-spatula where it spread on the water surface (2 mg on days 2–3, 4 mg on day 4, and 10 mg on day 5 until pupation). The solution feeding regime consisted of the same food quantity dissolved in deionised water (0.1 ml of 1 g/50 ml of TetraMin Baby on days 2–3, 0.2 ml of 1 g/50 ml of TetraMin Baby on day 4, and 0.5 ml of 1 g/50 ml of TetraMin Baby on day 5 until pupation) and injected into the larval pot using a pipette.

Mosquito larvae were reared in two water types with different levels of mineralisation: (i) deionised water, which was sourced from a reverse osmosis unit (PURELAB Prima, Wycombe, UK) installed in the laboratory. The water quality specifications of treated deionised water were: TDS (27.55 ± 2 mg/l), salinity (18.48 ± 1 ppm), conductivity (39.54 ± 2 µS), total organic carbon < 0.1 ppm, bacteria > 5 CFU/ml, 98% rejection of inorganics, > 99% rejection of organics, > 99% rejection of particles; (ii) mineral water containing natural minerals formed through geological processes and sourced in 5 l bottled from a local supplier. Water quality specifications for mineral water were: TDS (112.21 ± 2 mg/l); salinity (75.78 ± 1 ppm); conductivity (160.40 ± 2 µS) This water contained the following minerals per litre: calcium (11 mg); magnesium (3.5 mg); potassium (2.5 mg); sodium (10 mg); bicarbonate (25 mg); sulphate (11 mg); nitrate (15 mg); chloride (14 mg); dry residue at 180 °C (85 mg), and its pH was 6.2.

For each water type and feeding regime, mosquito larvae were reared in seven ammonia (NH_3_) concentrations, from 0 mg/l (control), 0.6 mg/l, 1.3 mg/l, 2.5 mg/l, 12.5 mg/l, 25 mg/l, and 62.5 mg/l, informed by published reports of the natural levels of ammonia in rice field flood water which are ≥ 0.5 mg/l and the toxicity level of unionised ammonia to freshwater aquatic organisms at concentrations > 0.2 mg/l [22–25, 32]. A stock solution of ammonium hydroxide equivalent to ~ 28–30% NH_3_ at 14.8 M (Sigma-Aldrich, St Louis, MO, USA) was diluted to 1% NH_3_ (~2.5 g/l) by adding a proportionate volume of water. Further dilutions were then made using the C_1_V_1_ = C_2_V_2_ dilution formula.

#### Developmental success parameters

Depending on the lifecycle stage of the mosquitoes, the following data were observed and recorded: (i) larval survival: determined as the percentage of larvae that developed into pupae from the total number of larvae for each treatment; (ii) pupal survival: determined as the percentage of mosquitoes that emerged as adults from those that pupated in each treatment; (iii) pupal mortality: determined as the percentage of mosquitoes that died at the pupal stage from the total number of mosquitoes per treatment; (iv) adult emergence: determined as the percentage of mosquitoes that emerged as adults from the total number of larvae in each treatment; (v) development time: determined as the number of days from placement of first instar larvae in treatment cups until adult emergence; (vi) wing length: following emergence, adult mosquitoes were sexed and stored in 75% ethanol. One wing of all emerged adults was measured from the distal end of the allula to the apical margin (radius veins), excluding the fringe scale using a binocular microscope. A stage micrometre of 1 mm ruler length (Graticules Ltd, Kent, UK) was used for calibration on 2.5× magnification on a scale of 1 microscope unit = 0.04 mm) [33].

### Experiment 2: developmental success and phenotypic quality of *An. gambiae* (s.s.) and *An. coluzzii* in contrasted microcosms

#### Experimental design

**Fig. 1:**
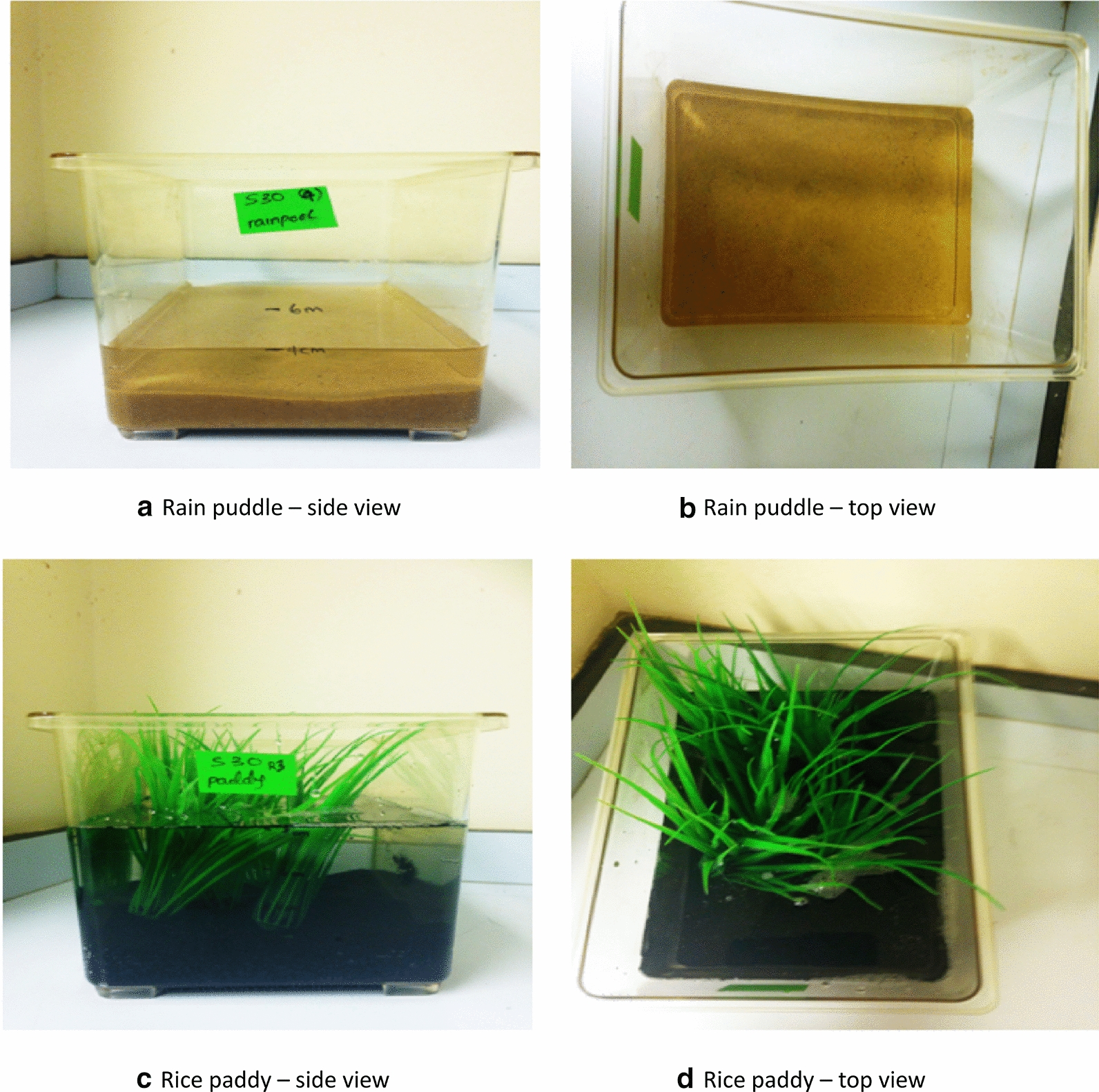
Divergent larval microcosms of the sibling species (Experiment 2). **a**,** b** Simulated *An. gambiae* (s.s.) preferred microcosm (rain puddle); **c**,** d** simulated *An. coluzzii*-typical larval habitat (rice paddy)

To investigate the developmental responses of the sibling species in contrasted larval habitats, first-instar larvae of *An. coluzzii* and *An. gambiae* (s.s.) were reared in rain puddle and rice paddy simulated microcosms. Rain puddle (*An. gambiae* (s.s.) preferred) larval microcosms were simulated with transparent aquarium-like containers of 19.5 cm length, 16.5 cm height, and 12.5 cm width, containing deionised water 4 cm deep and 400 ml of wet inert light brown sand (UNIPAC aqua gravel, Northampton, UK) of 2 cm depth (Fig. 1a, b). The rice paddy (*An. coluzzii* preferred) larval microcosm was simulated using a similar container, containing mineral water at 8 cm depth, 1.3 mg/l of ammonia, 400 ml of inert dark sand 2 cm deep (sourced as before), and a patch of intermediate density plastic grass (to imitate the rice field), 11.3 cm length × 6.2 cm width (patches were 5 cm apart) (Fig. 1c, d).

Each replicate consisted of two larval densities (30 and 60 larvae) of first-instar larvae of both species placed in the two types of microcosmic larval habitats (2 species × 2 densities × 2 habitats = 8 microcosms), resulting in a total of 720 sampled mosquito larvae for two replicates. The total of 16 microcosms was randomly arranged and rearranged daily from day 5 to avoid confounding effects. To prevent ammonia build-up, on day 5 and afterwards, every other day until the completion of the assay, water was pumped out from the microcosm using a low-pressure pump, down to a minimal level to avoid picking up larvae, and replaced with fresh water as per larval habitat type. Microcosms were covered with mosquito netting material to contain the emerging mosquito adults and entry of external debris. Coloured tapes were used to differentiate the habitat type and species, and the microcosm arrangement in the insectary was completely randomised (Additional file 2: Figure S2). All larvae were fed daily following a standardised feeding regime consisting of 0.1 μl of Liquifry liquid fish food (Interpret Ltd) on day 1. From day 2 until pupation, finely ground Tetramin flakes (Tetra GmbH) were mixed with deionised water and injected into the microcosm in the following proportions: day 2–3: 0.25 ml of 0.2 g/10 ml solution of ground Tetramin flakes; day 4: 0.5 ml of 0.16 g/10 ml solution of ground Tetramin flakes; day 5 until pupation: 1 ml of 0.75 g/50 ml of ground Tetramin flakes.

#### Developmental success parameters

Adult emergence was recorded as the number of adults that emerged from the total number of larvae placed in a microcosm. Development time was recorded as the duration between day 1 of the experiment when first instar larvae were placed in the microcosm and the day of adult emergence. Adults that emerged from the microcosms were collected using a mouth aspirator, sexed, and stored in 75% ethanol for subsequent measurement of wing length as described in the first experimental design.

#### Physicochemical water parameters

Levels of nitrates, ammonia, pH, general hardness, and carbonate hardness were measured using API aquarium test kits (Aquarium Pharmaceuticals, Mars Fishcare, Chalfont, PA, USA). Readings were taken 10 days after the experimental set-up for the first experiment (Table 1) and on days 1 and 10 for the second experiment (Table 2).

**Table 1:** Mean of nitrate, general hardness, carbonate hardness, pH, and ammonia (Experiment 1)

Water type	Ammonia	Nitrate (mg/l)	Ammonia (mg/l)	General hardness (mg/l)	Carbonate hardness (mg/l)	pH
Deionised	0	5	1	17.9	17.9	7.05
	0.6	5	3	17.9	17.9	7.05
	1.3	5	8	17.9	17.9	7.05
	2.5	5	–	17.9	35.8	7.2
	12.5	5	–	17.9	89.5	7.6
	25	–	–	–	–	–
Mineral	0	5	1.5	53.7	35.8	7.4
	0.6	5	2	53.7	35.8	7.6
	1.3	5	8	53.7	35.8	7.6+
	2.5	5	–	53.7	53.7	7.6 ++
	12.5	10	–	53.7	89.5	7.6+++
	25	10	–	35.8	125.3	7.6++++

Sample size *n* = 12 in all groups. Beyond pH 7.6, + show increasing darkening in colour change and pH of the solution without precise measurement of concentration

**Table 2:** Mean of general hardness, carbonate hardness, pH, nitrates, and ammonia across larval microcosms (Experiment 2)

Treatment	Species	Larval density	Day	Nitrate (mg/l)	Ammonia (mg/l)	General hardness (mg/l)	Carbonate hardness (mg/l)	pH
Rain puddle	*An. coluzzii*	30	1	0.5	0.25	17.9	17.9	6.8
			10	0	8	35.8	53.7	7.2
		60	1	0.5	0.25	17.9	17.9	6.8
			10	40	2.5	44.75	35.8	6.9
	*An. gambiae* (s.s.)	30	1	0.5	0.25	17.9	17.9	6.8
			10	0	4	35.8	35.8	6.8
		60	1	0.5	0.25	17.9	17.9	6.8
			10	40	0.75	35.8	35.8	6.6
Rice paddy	*An. coluzzii*	30	1	20	0.5	53.7	53.7	7.2
			10	0	4	71.6	53.7	6.7
		60	1	20	0.5	53.7	53.7	7.2
			10	0	8	71.6	53.7	7.2
	*An. gambiae* (s.s.)	30	1	20	0.5	53.7	53.7	7.2
			10	0	4	71.6	53.7	7.0
		60	1	20	0.5	53.7	53.7	7.2
			10	0	6	62.7	53.7	7.0

Sample size *n* = 2

### Statistical analysis

All data collected were analysed using the JMP 14 software (SAS Institute, Inc., Cary, NC, USA). All data were checked for deviations from normality and heterogeneity, and analyses were conducted using parametric and non-parametric methods as appropriate. Replicate effects were tested but are only reported when significant. Interactions between independent variables were tested using a stepwise approach whereby only those significant were retained in the final models. Following logistic regressions on proportions of larvae, pupae, and adults, likelihood odds ratios were used for *post-hoc* pairwise group comparisons. Body size, a continuous variable, was analysed through general linear models followed by Tukey’s HSD *post-hoc* pairwise comparisons. Finally, developmental times (day of emergence) were analysed by Cox proportional hazard models with likelihood odds ratios for *post-hoc* pairwise comparisons.

## Results

### Experiment 1: plastic response of *An. gambiae* (s.s.) and *An. coluzzii* in response to ammonium hydroxide exposure

#### Larval survival, pupal mortality, and emergence rates

**Fig. 2:**
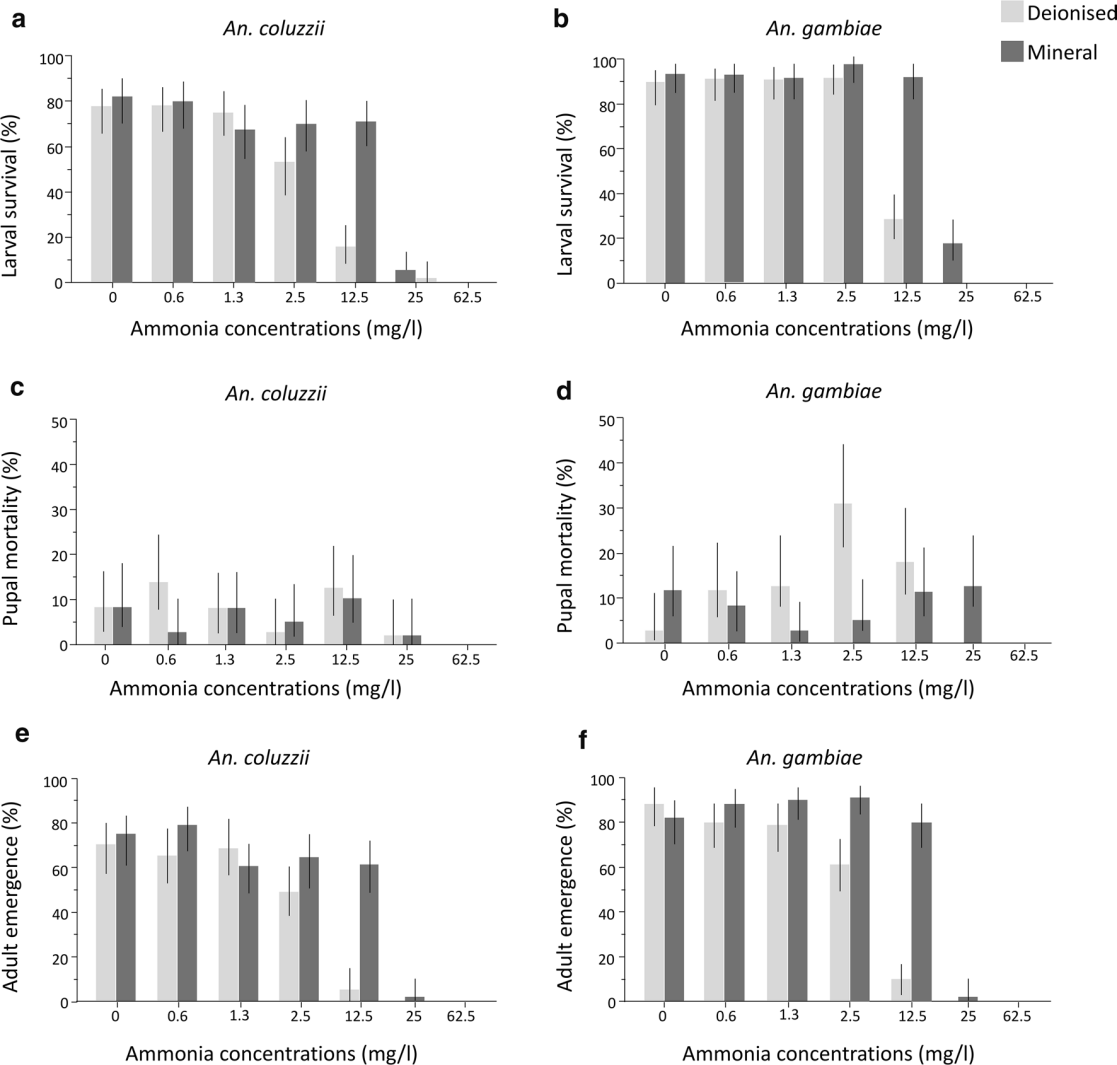
Mosquito survival with increasing ammonia concentrations across two water types (Experiment 1). Percentage survival at larval, adult stages, and mortality at pupal stage is shown for *An. coluzzii* (**a**, **e**, **c**) and *An. gambiae* (s.s.) (**b**, **f**, **d**). Light grey bars represent deionised water and dark grey bars mineral water. Whiskers represent 95% confidence intervals

Larval survival was strongly and significantly negatively affected by increasing ammonia concentration with numbers decreasing steadily from 1.3 mg/l to 25 mg/l ammonia and no larvae surviving at 62.5 mg/l (Tables 3, 4; Additional file 3: Figure S3). The logistic regression model also revealed that larval survival differed significantly between *An. gambiae* (s.s.) and *An. coluzzii* with the former having on average a 14% higher larval survival (Tables 3, 4; Fig. 2a, b). Additionally, larval survival was significantly impacted by water types with survival 12% higher in mineralised water compared to deionised water (Tables 3, 4; Fig. 2a, b). There was a significant interaction between water type and ammonia highlighting the fact that survival decreased with ammonia concentrations much faster in deionised water (Table 4; Fig. 2a, b). Additionally, increasing ammonia concentrations impacted *An. coluzzii* more severely than *An. gambiae* resulting in a significant species × ammonia interaction (Table 4; Fig. 2a, b).

**Table 3:** Effect of ammonia, water types, and feed regimes on life history stages (Experiment 1)

Species	Water type	Feed regime	Ammonia (mg/l)	% Larval survival	% Pupal mortality	% Adult emergence
*An. coluzzii*	Deionised	Solution	0	77 (59–88)	10 (3–26)	67 (49–81)
			0.6	73 (56–86)	0	73 (56–86)
			1.3	70 (52–83)	13 (5–30)	57 (39–73)
			2.5	57 (39–73)	3 (1–17)	53 (36–70)
			12.5	33 (19–51)	23 (12–41)	10 (3–26)
			25	3 (0–17)	3 (0–17)	0
			62.5	0	0	0
		Powder	0	77 (59–88)	3 (1–17)	73 (56–86)
			0.6	83 (66–93)	27 (14–44)	57 (39–73)
			1.3	80 (63–90)	0	80 (63–90)
			2.5	50 (33–67)	3 (1–17)	47 (30–64)
			12.5	0	0	0
			25	0	0	0
			62.5	0	0	0
	Mineral	Solution	0	80 (63–90)	7 (2–21)	73 (56–86)
			0.6	80 (63–90)	3 (1–17)	77 (59–88)
			1.3	73 (56–86)	10 (3–26)	63 (46–78)
			2.5	67 (49–81)	3 (1–17)	63 (46–78)
			12.5	73 (56–86)	3 (1–17)	70 (52–83)
			25	10 (3–26)	3 (1–17)	7 (2–21)
			62.5	0	0	0
		Powder	0	83 (66–93)	10 (3–26)	73 (56–86)
			0.6	80 (63–90)	3 (1–17)	77 (59–88)
			1.3	60 (42–75)	3 (1–17)	57 (39–73)
			2.5	73 (56–86)	7 (2–21)	67 (49–81)
			12.5	70 (52–83)	17 (7–34)	53 (36–70)
			25	0	0	0
			62.5	0	0	0
*An. gambiae* (s.s.)	Deionised	Solution	0	86 (70–95)	7 (2–21)	80 (63–90)
			0.6	97 (83–99)	10 (3–26)	87 (70–95)
			1.3	90 (74–97)	0	90 (74–97)
			2.5	87 (70–95)	17 (13–34)	70 (52–83)
			12.5	43 (27–61)	23 (12–41)	20 (10–37)
			25	0	0	0
			62.5	0	0	0
		Powder	0	93 (79–98)		93 (79–98)
			0.6	87 (70–95)	13 (5–30)	73 (56–86)
			1.3	93 (79–98)	27 (14–44)	67 (49–81)
			2.5	100(89–100)	47 (30–64)	53 (36–70)
			12.5	13 (5–30)	13 (5–30)	0
			25	0	0	0
			62.5	0	0	0
	Mineral	Solution	0	93 (79–98)	17 (7–34)	77 (59–88)
			0.6	97 (83–99) 30	10 (3–26) 30	87 (70–95) 30
			1.3	87 (70–95)	3 (1–17)	83 (66–93)
			2.5	100(89–100)	0	100(89–100)
			12.5	93 (79–98)	10 (3–26)	83 (66–93)
			25	13 (5–30)	7 (2–21)	7 (2–21)
			62.5	0	0	0
		Powder	0	93 (79–98)	7 (2–21)	87 (70–95)
			0.6	90 (74–97)	3 (1–7)	87 (70–95)
			1.3	97 (83–99)	0	97 (83–99)
			2.5	93 (79–98)	10 (3–26)	83 (66–93)
			12.5	90 (74–97)	13 (5–30)	77 (59–88)
			25	20 (10–37)	20 (10–37)	0
			62.5	0	0	0

Ninety-five percent confidence intervals are in parentheses. Larval survival, pupal mortality, and emergence rates were calculated out of an initial sample size of 30 larvae (per treatment)

**Table 4:** Logistic regressions of the effect of ammonia, water types, and feed regimes on life history stages (Experiment 1)

Parameter	Source	*df*	Likelihood ratio	*P*-value
Larval survival	Species	1	20.614	< 0.0001***
	Water type	1	78.060	< 0.0001***
	Feed	1	1.565	0.2109^ns^
	Ammonia	1	1072.061	< 0.0001***
	Ammonia * Water type	1	31.720	< 0.0001***
	Ammonia * Species	1	7.325	0.0068**
Pupal mortality	Species	1	8.062	0.0045**
	Water type	1	4.328	0.0375*
	Feed	1	1.286	0.2567^ns^
	Ammonia	1	32.472	< 0.0001***
Adult emergence	Species	1	37.496	< 0.0001***
	Water type	1	92.267	< 0.0001***
	Feed	1	3.872	0.0491*
	Ammonia	1	941.705	< 0.0001***
	Ammonia * water type	1	43.053	< 0.0001***

^***^P < 0.0001; ** *P* < 0.01; *P < 0.05; ^ns^ > 0.05

*df*, degrees of freedom

Pupal mortality also significantly increased with ammonia concentrations (Tables 3, 4; Fig. 2c, d; Additional file 4: Figure S4). There was a significant difference in pupal mortality between the sibling species with 4% higher mortality in *An. gambiae* (s.s.) compared to *An. coluzzii* (Tables 3, 4; Fig. 2c, d). Water type significantly impacted pupal mortality, which was 2% higher in deionised water compared to mineral water (Tables 3, 4; Fig. 2c, d). Feed regime had no significant impact on pupal mortality, and there was no interactive effect between increasing ammonia concentrations and other variables (Table 4; Additional file 4: Figure S4).

Overall, the logistic regression model on adult emergence revealed a significant decline in mosquito survival with increasing ammonia concentrations (Tables 3, 4; Additional file 5: Figure S5). Percentage of adult emergence declined significantly in a stepwise fashion from concentrations > 2.5 mg/l to 25 mg/l with 100% mortality at 62.5 mg/l (Table 4; Additional file 5: Figure S5). Additionally, water types significantly impacted adult emergence with 14% more adults emerging from mineral water compared to deionised water. The significant interaction between ammonia and water type resulted in increasingly higher adult emergence rates in mineral water compared to deionised water (Table 4; Fig. 2e, f). For instance, at 2.5 mg/l and 12.5 mg/l ammonia concentrations, adult emergence was 23% and 63% higher, respectively, in mineral water compared to deionised water, and at 25 mg/l, adults emerged only from the mineral water (Tables 3, 4; Fig. 2e, f). There was a significant impact of species on adult emergence with *An. gambiae* (s.s.) having 10% higher adult emergence compared to *An. coluzzii* (Tables 3, 4; Fig. 2e, f). Feed regimes significantly impacted adult emergence (Table 4) with emergence 4% higher in solution feed compared to powder feed (Table 3; Additional file 5: Figure S5).

#### Sex ratio

Overall, the proportion of female emergence in this experiment was 44% (Table 5). Logistic regression revealed that the sex ratio of emerging mosquitoes was significantly impacted by species; male emergence was 20% higher and female emergence > 1% higher in *An. gambiae* (s.s.) compared to *An. coluzzii* (Table 6). Water type also had a significant impact on the sex ratio, with 30% more females and 8% more males surviving in mineral water compared to deionised water (Table 6). Feed regime and increasing ammonia concentration had no significant impact on sex ratio.

**Table 5:** Goodness-of-fit test (likelihood ratio) of mosquito equal sex ratio across species and water types (Experiment 1)

Species	Water types	Female proportion (%)	Sample size	Chi-square	*df*	*P*-value
*An. coluzzii*	Deionised	45 (37–52)	155	1.8683	1	0.1717^ns^
	Mineral	53 (46–60)	204	0.7063	1	0.4007^ns^
*An. gambiae* (s.s.)	Deionised	37 (30–44)	190	13.3141	1	0.0003**
	Mineral	43 (37–49)	260	5.5738	1	0.0182*

^***^P < 0.0001; **P < 0.01; *P < 0.05; ^ns^ > 0.05. Ninety-five percent confidence intervals are in parentheses

**Table 6:** Logistic regression (effect likelihood ratio tests) on the sex ratio of *An. coluzzii* and *An. gambiae* (s.s.) at emergence (Experiment 1)

Source	*df*	Likelihood ratio	*P-*value
Species	1	6.920	0.0085*
Water type	1	3.908	0.0480*
Feed	1	3.431	0.0640^ns^
Ammonia	1	0.005	0.9436^ns^

^***^P < 0.0001; ** *P* < 0.01; *P < 0.05; ^ns^ > 0.05

Likelihood ratio tests of probabilities revealed significant deviations from a 50:50 male-female ratio in *An. gambiae* (s.s.) survival, with lower female emergence in both water types (Table 5). There was no significant deviation from the 50:50 ratio in *An. coluzzii*. The percentage of female survival of *An. gambiae* (s.s.) was lower in deionised water (37%) compared to mineral water (43%) (Table 5). Similarly, female survival in *An. coluzzii* was 8% lower in deionised water compared to mineral water (Table 5).

#### Adult body size

**Fig. 3:**
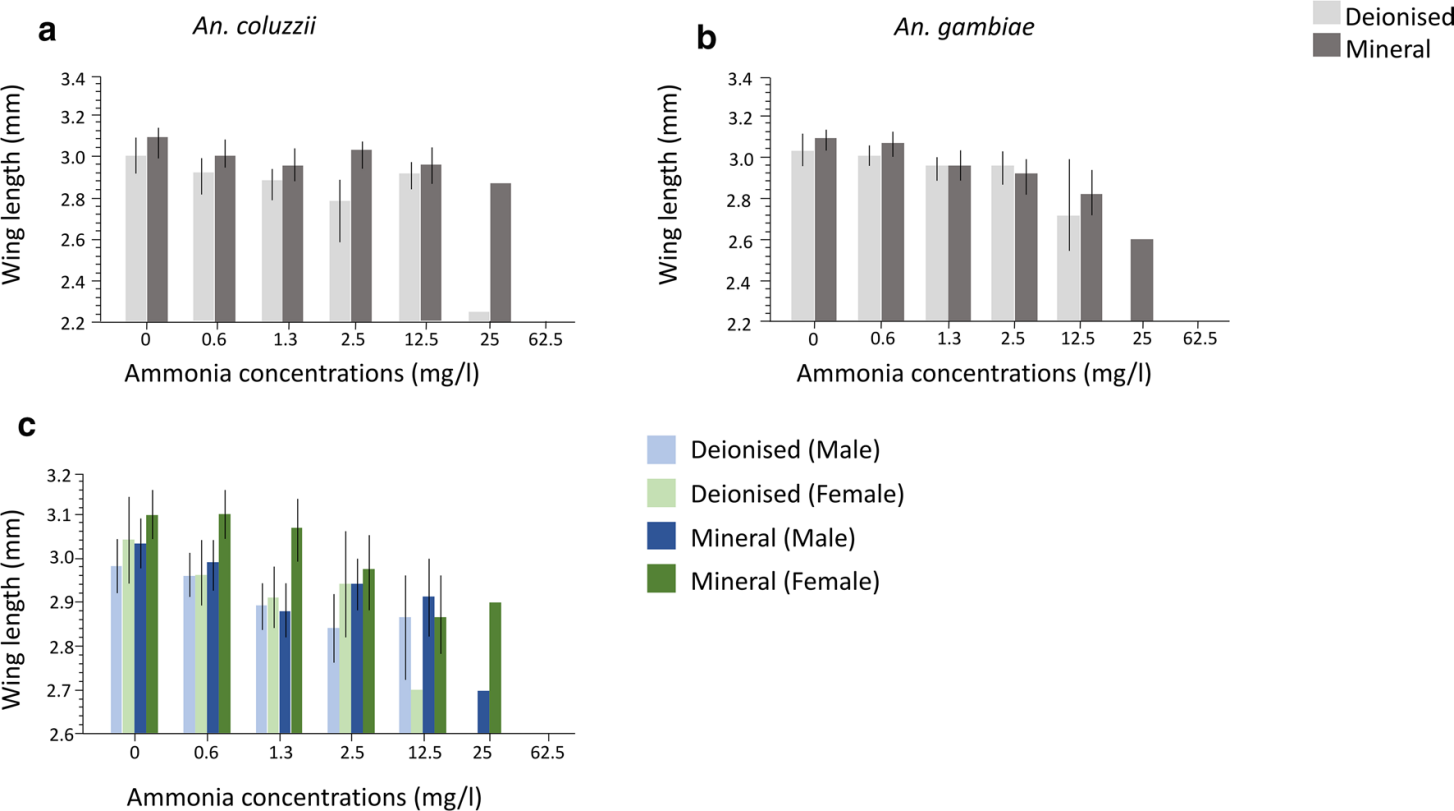
Effect of ammonia and water types on wing length (Experiment 1). The mean wing length for *An. coluzzii* (**a**) and *An. gambiae* (s.s.) (**b**) shown for two water types (light grey bars, deionised; dark grey bars, mineral). **c** Mean wing length of adult males in deionised water (light blue), mineral water (dark blue) and females in deionised water (light green), mineral water (dark green). Whiskers represent 95% confidence intervals

There was a significant decrease in adult body size (wing length) with increasing ammonia concentrations (Table 7; Fig. 3; Additional file 6: Table S1). The general linear model also revealed that although there was no direct effect of species on adult body size, the negative impact of ammonia differed between species resulting in significant G × E interactions of species with ammonia. In *An. coluzzii* imagoes this translated into larger body size with increasing ammonia concentrations compared to *An. gambiae* (s.s.) (Table 7; Fig. 3; Additional file 6: Table S1). For instance, above 2.5 mg/l, *An. coluzzii* adults were larger than *An. gambiae* (s.s.) adults (Fig. 3a, b). Water type significantly impacted body size, with adults emerging from mineral water significantly bigger than those from deionised water (Table 7; Fig. 3a, b). Feed regimes did not have a significant impact on wing length (Table 7). Wing length was significantly impacted by sex, with females on average significantly larger than males (Table 7; Fig. 3c). The significant interaction between sex and ammonia concentration highlighted the fact that increasing ammonia concentrations had a stronger negative effect on males than females (Table 7; Fig. 3c).

**Table 7:** General linear model of the effect of ammonia, water types, and feed regimes on wing length (Experiment 1)

Parameter	Source	*df*	*F*-ratio	*P-*value
Wing length	Species	1	3.1706	0.0754^ns^
	Feed	1	0.4523	0.5014^ns^
	Ammonia	1	30.6251	< 0.0001***
	Water type	1	15.8197	< 0.0001***
	Sex	1	11.3657	0.0008**
	Sex * Ammonia	1	5.2245	0.0225*
	Ammonia * Species	1	6.6604	0.0100*
	Water type * Species	1	6.9135	0.0087*

^***^P < 0.0001; ** *P* < 0.01; *P < 0.05; ^ns^ > 0.05

*df*, degrees of freedom

#### Developmental time

**Fig. 4:**
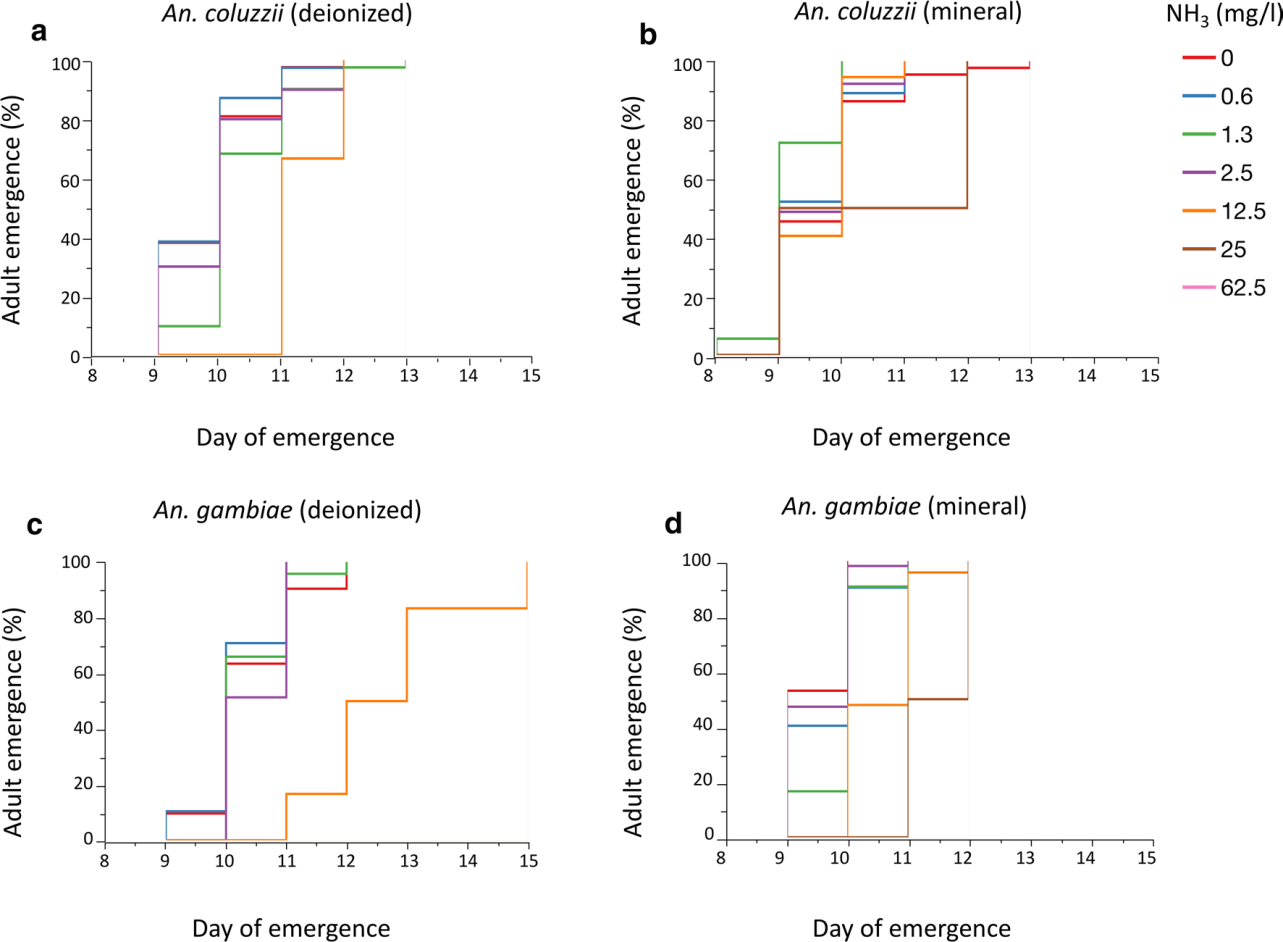
Effect of ammonia and water types on development time (Experiment 1). Mean development time for *An. coluzzii* (**a**, **b**) and *An. gambiae* (s.s.) (**c**, **d**) reared in deionised and mineral water across seven concentrations of ammonia

The Cox proportional hazard model revealed that the duration of development from first-instar larvae to adult also increased with increasing ammonia concentrations (Table 8; Fig. 4; Additional file 6: Table S1). There was a significant effect of species on development time which was significantly longer in *An. gambiae* (s.s.) compared to *An. coluzzii* (Table 8). The significant G × E interaction between species and ammonia resulted in longer development time for *An. gambiae* (s.s.) at higher ammonia concentrations (Table 8). For instance, above 12.5 mg/l, *An. coluzzii* emerged 1 day earlier than *An. gambiae* (s.s.) (Table 8; Fig. 4). Water type significantly impacted development time as mosquitoes reared in deionised water took longer to complete their development cycle compared to mineral water (Table 8; Fig. 4; Additional file 6: Table S1).

**Table 8:** Cox proportional hazard analyses of the effect of ammonia, water type, and feed on development time (Experiment 1)

Parameter	Source	*df*	Wald chi-square	*P*-value
Day of emergence	Species	1	9.272	0.0023**
	Water type	1	48.369	< 0.0001***
	Feed	1	0.607	0.4361^ns^
	Ammonia	1	19.552	< 0.0001***
	Ammonia * Species	1	5.74302292	0.0166*

***P < 0.0001; ** *P* < 0.01; *P < 0.05; ^ns^ > 0.05

*df*, degrees of freedom

### Experiment 2: contrasted microcosm experiment and developmental success reaction norms in *Anopheles gambiae* (s.s.) and *Anopheles coluzzii*

#### Emergence rates

**Fig. 5:**
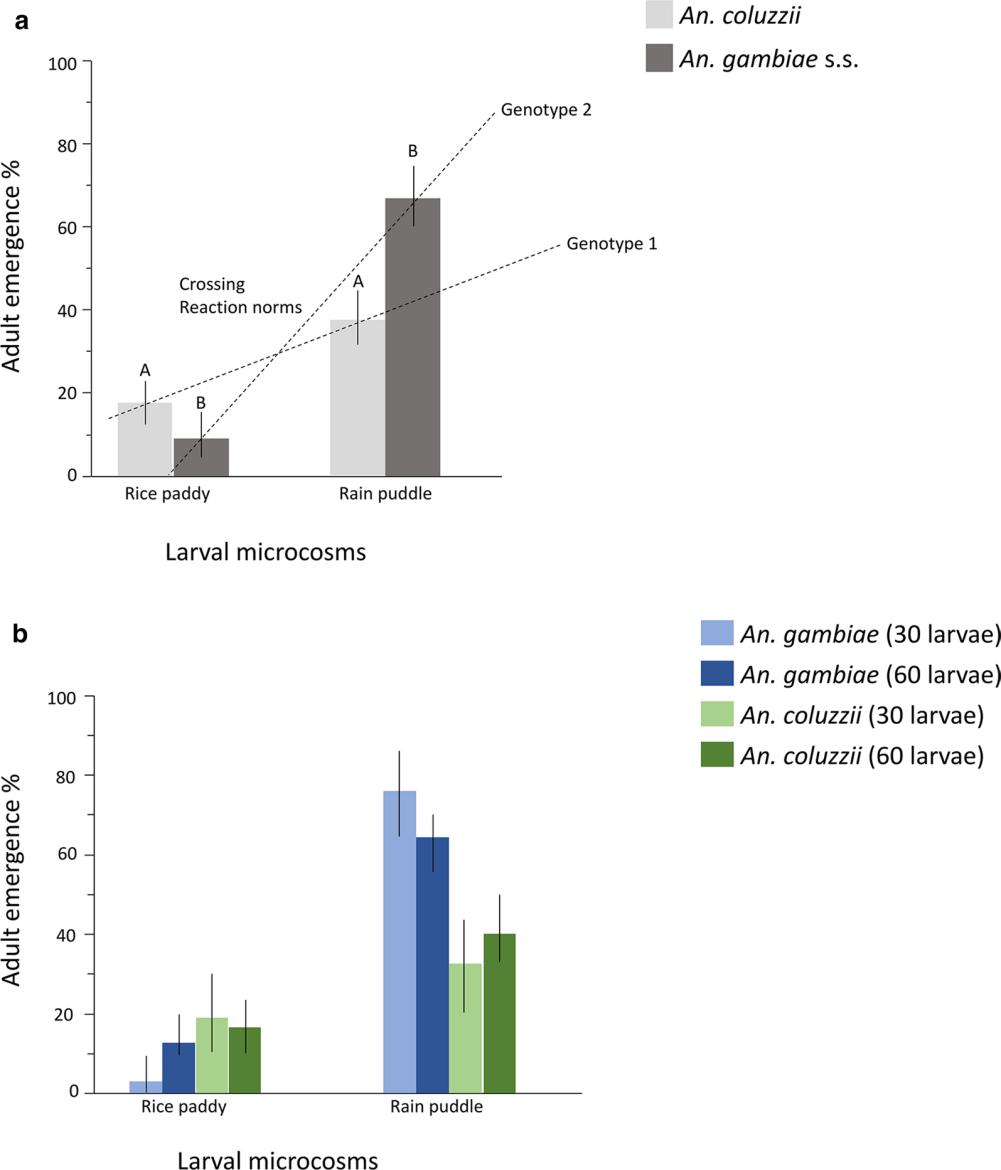
Adult emergence of across divergent larval microcosms (Experiment 2). Mean adult emergence is shown for *An. coluzzii* (**a**) (light grey bars) and *An. gambiae* (s.s.) (dark grey bars); within larval microcosms, significant differences are represented by different letters. The two overlaid dashed lines highlight the crossing G × E reaction norms across two divergent larval environments. **b** Mean adult emergence is shown for *An. gambiae* (s.s.) (light blue, 30; dark blue, 60) and *An. coluzzii* (light green, 30; dark green, 60) at two larval densities. Whiskers represent 95% confidence intervals (CI)

The logistic regression model revealed a significant effect of microcosm type on adult emergence success (Table 9), with an overall 40% higher emergence from rain puddle compared to rice paddy. Although there was no significant direct impact of species on adult emergence, there was a strong and significant G × E interaction between species and microcosm on adult emergence (Table 9). In the rice paddy microcosm, adult emergence for *An. coluzzii* was 17%, almost double the 9% of *An. gambiae* (s.s.). Conversely, in the rain puddle microcosm, 67% of *An. gambiae* emerged compared to 38% in *An. coluzzii*. (Fig. 5a; Additional file 7: Table S2). Larval density had no significant impact on adult emergence (Table 9; Fig. 5b).

**Table 9:** Logistic regression (effect likelihood ratio tests) of microcosm and density on adult emergence success of *An. coluzzii* and *An. gambiae* (s.s*.*) (Experiment 2)

Source	df	Likelihood ratio	*P*-value
Species	1	0.338	0.1701^ns^
Microcosm	1	60.481	< 0.0001***
Density	1	2.646	0.7013^ns^
Microcosm * Species	1	28.886	< 0.0001***

***P < 0.0001; **P < 0.01; *P < 0.05; ^ns^ > 0.05

*df* degrees of freedom

#### Sex ratio

Overall, there was a 54% female emergence rate in the second experiment (Table 10). Logistic regression revealed that there was no significant impact of species, microcosm, and density on the sex ratio of emerged adults (Table 11). Similarly, likelihood ratio tests (50:50) of probabilities revealed no significant deviations in the sex ratio for both species across microcosms (Table 10).

**Table 10:** Goodness-of-fit test (likelihood ratio) of mosquito sex ratio across microcosms (Experiment 2)

Species	Microcosm	Female proportion (%)	Sample size	Chi-square	*df*	*P*-value
*An. coluzzii*	Rice paddy	53 (36–70)	30	0.1334	1	0.7149^ns^
	Rain puddle	57 (45–68)	69	1.1773	1	0.2779^ns^
*An. gambiae* (s.s.)	Rice paddy	41 (22–64)	17	0.5322	1	0.4657^ns^
	Rain puddle	54 (45–62)	121	0.6700	1	0.4130^ns^

^***^P < 0.0001; ** *P* < 0.01; *P < 0.05; ^ns^ > 0.05. Ninety-five percent confidence intervals are in parentheses

**Table 11:** Logistic regression (effect likelihood ratio tests) of development success of the sibling species by sex (Experiment 2)

Source	*df*	Likelihood ratio	*P*-value
Species	1	0.503	0.4780^ns^
Microcosm	1	0.640	0.4238^ns^
Density	1	0.531	0.4660^ns^

^***^P < 0.0001; ** *P* < 0.01; *P < .05; ^ns^ > 0.05

#### Body size

**Fig. 6:**
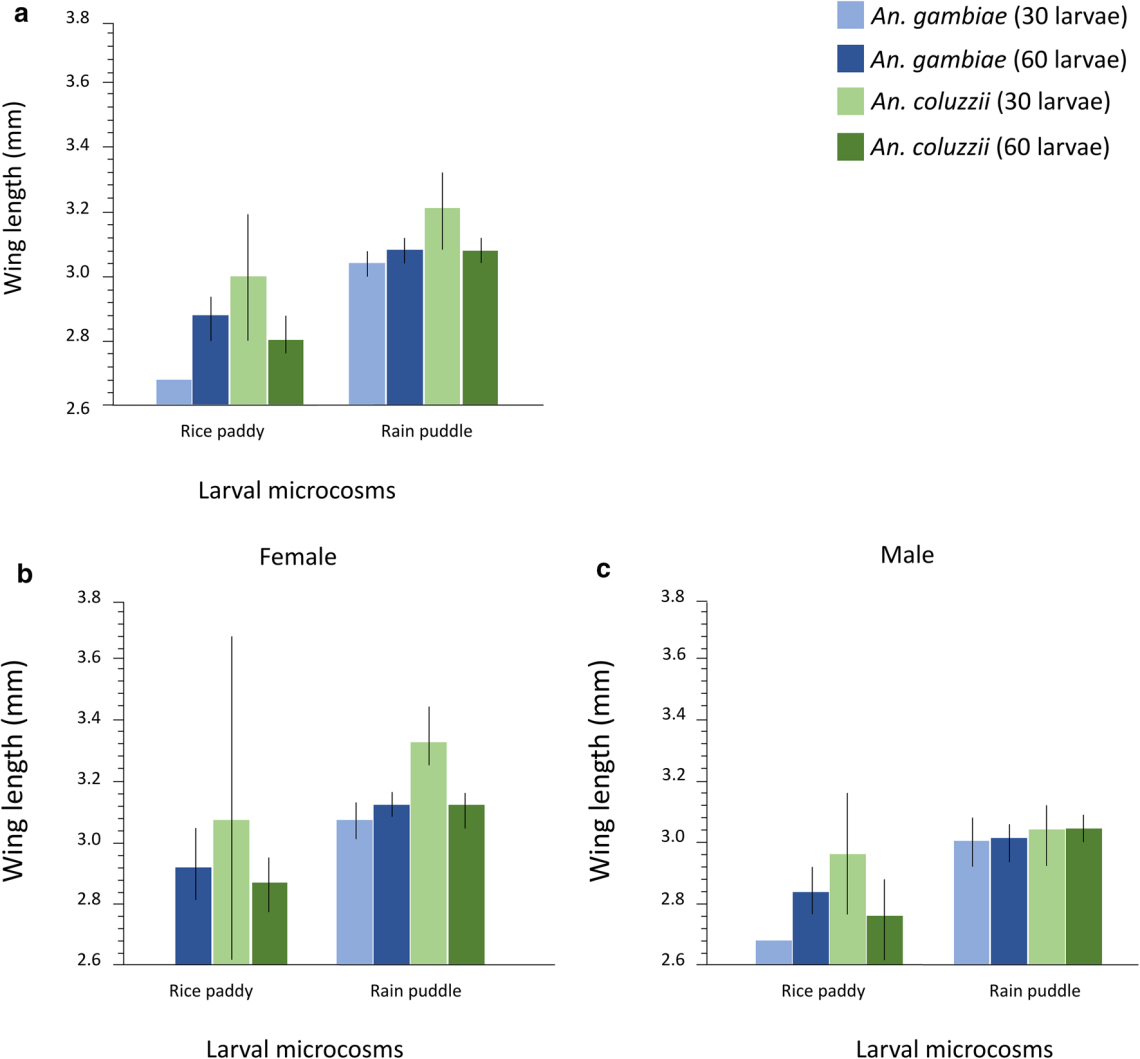
Mean wing length of the sibling species across microcosms (Experiment 2). **a** Wing length of *An. gambiae* (light blue, 30 larval density; dark blue, 60 larval density) and *An. coluzzii* (light green, larval density of 30; dark green, larval density of 60) across microcosms. **b**, **c** Wing length by sex comparison of *An. gambiae* (s.s.) (light blue, larval density of 30; dark blue, larval density of 60) and *An. coluzzii* (light green, larval density of 30; dark green, larval density of 60) across microcosms. Whiskers represent 95% confidence intervals

Microcosm type had a significant impact on adult body size as adults emerging from the rain puddle microcosm were bigger than those from the rice paddy larval microcosm (Table 12; Fig. 6a; Additional file 8: Table S3). There was a significant effect of species on adult body size as emerging *An. coluzzii* adults were on average bigger than *An. gambiae* (s.s.) adults (Table 12; Fig. 6a; Additional file 8: Table S3). Adult body size was also impacted by larval rearing density with imagoes reared at 30 larvae per microcosm significantly bigger than at 60 larvae rearing density (Table 12; Fig. 6a; Additional file 8: Table S3). The effect of density significantly interacted with species (Table 12). *Post-hoc* pairwise comparisons revealed that for both rearing densities (30 and 60), *An. coluzzii* adults had significantly longer wing lengths compared to *An. gambiae* (Tukey’s HSD test: *t*-ratio ≥ 4.24; *P* ≤ 0.0002 in both cases) (Fig. 6a; Additional file 8: Table S3). Adult body size was significantly impacted by sex with emerged females bigger than the males (Fig. 6b, c; Table 8).

**Table 12:** General linear model of the effect of microcosm and density on wing length across larval microcosms (Experiment 2)

Parameter	Source	*df*	*F*-ratio	*P*-value
Wing length	Species	1	28.707	< 0.0001***
	Microcosm	1	81.735	< 0.0001***
	Density	1	5.766	0.0171*
	Sex	1	30.593	< 0.0001***
	Density*Species	1	21.548	< 0.0001***

^***^P < 0.0001; ** *P* < 0.01; *P < 0.05;

*df*, degrees of freedom

Further linear regression models conducted for each sex separately revealed significant effects of species, microcosm, density, and species * density on the wing length of females in the same direction as the main model (Table 13; Fig. 6b, c). Whilst species significantly impacted adult male body size with *An. coluzzii* males being larger than *An. gambiae* (s.s.) males, microcosm type and rearing density had no significant impact on body size in males (Table 13; Fig. 6b, c; Additional file 8: Table S3).

**Table 13:** General linear models of the effect of microcosm and density on wing length by sex (Experiment 2)

Parameter	Sex	Source	*df*	*F*-ratio	*P*-value
Wing length	Female	Species	1	26.573	< 0.0001***
		Microcosm	1	39.858	< 0.0001***
		Density	1	5.973	0.0160*
		Density*Species	1	20.869	< 0.0001***
	Male	Species	1	1.772	0.1860^ns^
		Microcosm	1	34.769	< 0.0001***
		Density	1	0.208	0.6491^ns^

^***^P < 0.0001; ** *P* < 0.01; *P < 0.05; ^ns^ > 0.05.

*df*, degrees of freedom

#### Developmental time

**Fig. 7:**
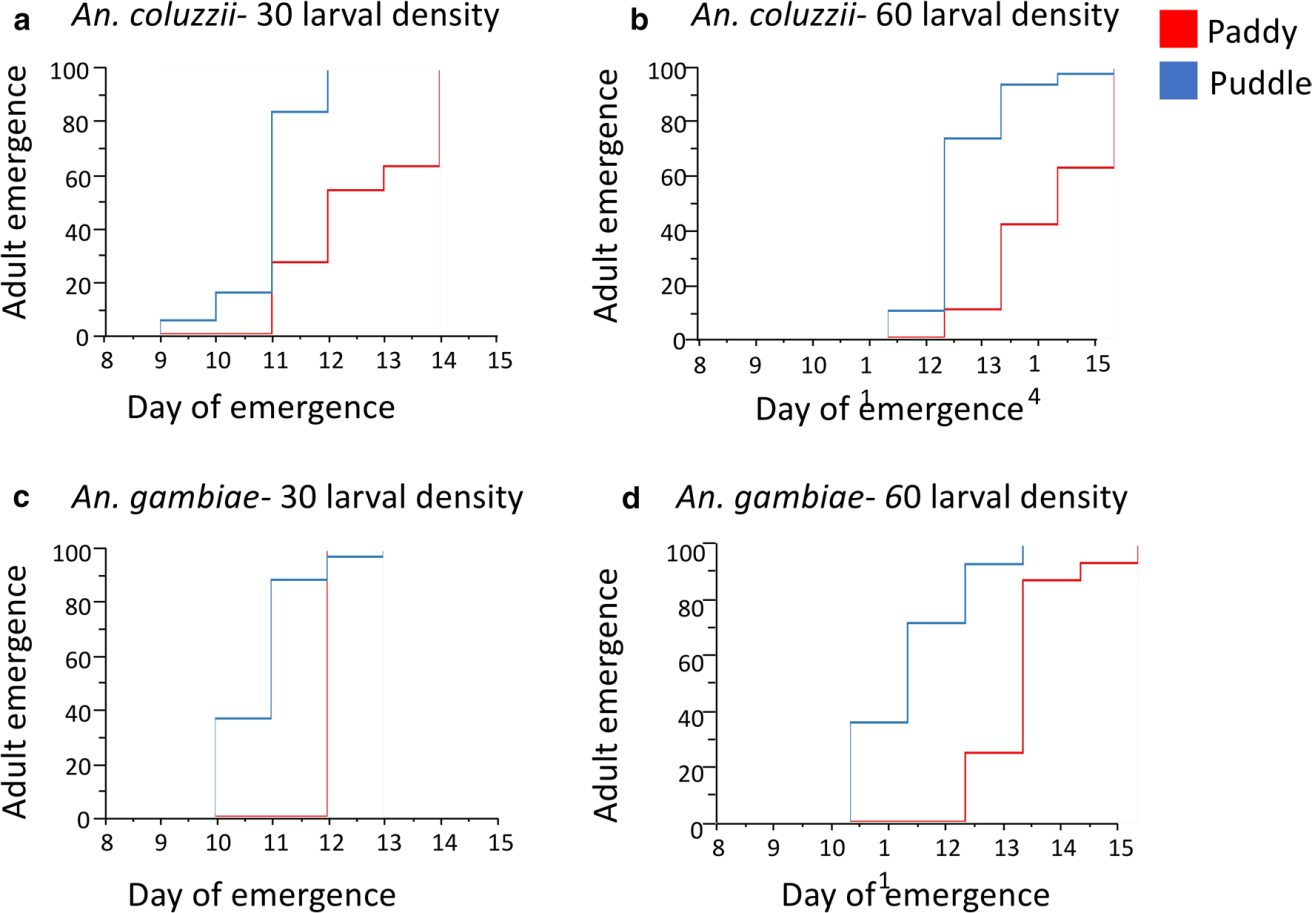
Development time across larval microcosms (Experiment 2). Mean development time for *An. coluzzii* (**a** larval density of 30 and **b** larval density of 60) and *An. gambiae* (**c** larval density of 30 and **d** larval density of 60)

Cox proportional hazard model showed that microcosm significantly impacted development time, which was on average 2 days longer in the rice paddy microcosm compared to the rain puddle microcosm (Table 14; Fig. 7; Additional file 8: Table S3). There were no significant effects of species and density on the duration of mosquito development (Table 14).

**Table 14:** Cox proportional hazard analyses of the effect of microcosm and density on development time (Experiment 2)

Parameter	Source	*df*	Likelihood ratio	*P*-value
Day of emergence	Species	1	1.467	0.2258^ns^
	Microcosm	1	41.917	< 0.0001***
	Density	1	2.759	0.0967^ns^

^***^P < 0.0001; ** *P* < 0.01; *P < 0.05; ^ns^ > 0.05

*df*, degrees of freedom

## Discussion

This laboratory study highlights, for the first time, contrasted plastic species-specific responses to ammonia and mineralisation, both characteristic of more eutrophic and permanent habitats used by *An. gambiae* (s.s.) and *An. coluzzii* [24, 34, 35]. In the microcosm-based experiment, the sibling species exhibited plastic responses in developmental success and adult phenotypic quality that matched those expected given their larval habitat preferences in nature [2, 6, 36]. As predicted, *An. coluzzii* had higher survival in the rice paddy environment with emerging adults of superior phenotypic quality compared to *An. gambiae* (s.s.), and *vice versa*, in the rain puddle environment. This study therefore successfully created larval rearing conditions eliciting G × E interactions akin to those found in the natural habitats of these important malaria vector sibling species. These results lend further support to the hypothesis of ecological speciation through larval adaptation to rice domestication by *An. coluzzii* [18, 37]. The crossing reaction norms observed in this study highlight a strong G × E interaction driven by the combination of ammonia, mineralisation and further exacerbated by water depth. Crisscrossing reaction norms highlight a simple process of contrasted species-specific adaptations and resulting selection responses that can promote further ecological divergence [13].

In the first experiment, the higher survival of *An. gambiae* (s.s.) at high NH_3_ concentrations was unexpected as *An. coluzzii*, the more recently derived taxon, had previously been shown to have more tolerance for ammonia [27]. One possible explanation is that the longer adaptation of *An. gambiae* (s.s.) to standard insectary rearing conditions (~ 40 years of laboratory colonisation compared to the ~ 17-year-old *An. coluzzii* strain) negated any advantage that *An. coluzzii* might have had in terms of ammonia tolerance. Interestingly, we found that *An. coluzzii* had larger adult body size compared to the *An. gambiae* (s.s.) strain at higher ammonia concentrations—this was independent of nutrition, as the food was provided *ad libitum*. Adult mosquito size measured as wing length is an important phenotypic trait in *Anopheles* mosquitoes [38]. Larger body size in *An. gambiae* (s.l.) has been shown to depend on optimal larval growth conditions [39, 40]. In turn, larger adults have been shown to have higher fitness, with a longer intrinsic lifespan, increased ability to withstand stress, higher male mating success, and increased female fecundity [4, 41].

Although the first experiment did not reveal contrasted reaction norms in development success among the sibling species to ammonia, it did reveal significant effects of the interaction of ammonia and water mineralisation on the developmental success and body size of *An. gambiae* (s.s.) and *An. coluzzii*. First, larval survival, pupal survival, and adult emergence were significantly higher in mineral water compared to deionised water. This supports published evidence that the ammonia toxicity risk of elevated pH and temperature can be greatly reduced with alkaline buffer > pH 8 [23, 24]. Mineral water used in the study had a mean general hardness of 53.7 mg/l compared to the 17.9 mg/l of deionised water, resulting in higher pH values above the minimum threshold where the buffering effect was activated [23, 25]. The results of this study clearly show that the presence of minerals in the water constituted a buffer that resulted in the reduction of the impact of ammonia toxicity, especially at higher ammonia concentrations. Second, adults emerging from mineral water were significantly bigger than those from deionised water. Likewise, development time for both species was significantly longer in deionised water compared to mineral water. These results further strengthen the argument for the buffering capacity of mineral water to reduce the effect of ammonia on aquatic organisms, resulting in the higher percentage survival and better adult quality of *An. gambiae* (s.s.) and *An. coluzzii* [23, 24, 42].

We also found that the proportion of females at emergence was lower in deionised water for both species, lending further credence to the suitability of minerals for rearing these species [42]. The effect was more severe in *An. gambiae* (s.s.) females in both water types compared to *An. coluzzii*. As we had never observed such bias in sex ratio under our standard rearing protocol which uses 2 cm of water in rearing trays, we hypothesised that water depth could have been an important parameter leading to the observed bias in sex ratio. In nature, the water puddles favoured by *An. gambiae* tend to be shallower than more permanent breeding sites [20, 31, 43].

Following on from the results of the first experiment, water mineralisation, ammonia, and water depth were identified as key environmental factors of larval breeding sites that can differentially impact the developmental success of the sibling species. This informed the design of the second experiment, whereby these factors were combined by creating contrasted microcosms allowing them to interact together to reveal a possible species × environment reaction norm. As expected, the second experiment revealed significant and crossing reaction norms in the developmental success and phenotypic quality of the sibling species in response to the two contrasting sets of environmental conditions. *An. coluzzii* had significantly higher survival in the rice paddy microcosm compared to *An. gambiae* and *vice versa* in rain puddle. The environmental conditions in the rice paddy microcosm consisted of higher mineralisation and ammonium levels and higher water depth. All of these interacted in a way that revealed the ability of *An. coluzzii* to exploit more successfully eutrophic permanent larval breeding sites [2, 6, 18, 36, 37]. In contrast, *An. gambiae* (s.s.) was more successful in the lower mineralisation, shallow depth, rain puddle microcosm. These contrasting reaction norms revealed pre-existing genetic traits and development of organismal homeostasis in *An. coluzzii*, but this time it also positively impacted survival, not just adult body size as in the first experiment [18]. These significant differences in developmental success between the sibling species within different larval habitats resulted in crossing reaction norms, with very strong G × E interaction, thus, providing strong evidence for ecological speciation. The results were reflective of the adaptive response and higher tolerance of *An. coluzzii* to ammonia in its larval habitat, which in the context of a scenario of peripatric speciation with gene flow may have been the most important driver of divergence from the ancestral *An. gambiae* (s.s.) [2, 6, 12, 36, 44]. These results were also consistent with field-obtained data on the sibling species. *Anopheles coluzzii* prefer permanent, organically rich, predator-prone habitats, whereas its sibling species, *An. gambiae* (s.s.), prefers temporal, shallow, rain-fed pools [2, 6, 12, 36]. Additionally, as in the first experiment, survival of *An. gambiae* (s.s.) females was more negatively impacted by water depth in the rice paddy microcosm, a reaction norm that could be indicative of adaptation to shallow water, unlike *An. coluzzii*, which is better adapted to deeper, more permanent larval breeding sites like rice paddies [43].

Following a similar reaction norm as in the first experiment, *An. coluzzii* adults were significantly larger than *An. gambiae* (s.s.) across both microcosms. Previous studies have amply demonstrated that larval growth conditions influence adult body size in *An. gambiae* (s.l.) [39–41]. The results from this study support field and laboratory studies on *An. coluzzii* and *An. gambiae* and strengthen the theory of divergent adaptations in their larval habitat [2, 6, 12, 36]. The *An. coluzzii* strain reared in CAEP, Keele Laboratory, appears to have maintained the reaction norms developed presumably from adaptation to rice field habitats over the years in their ability to adapt to similar rearing conditions. Higher phenotypic quality evidenced in their larger body size compared to *An. gambiae* across both larval habitats is indicative of a crossing reaction norm linked to ammonia tolerance [10].

The contrasted slopes of reaction norms to ammonia also exemplify why and how sympatric sibling species can be found in the same region despite imperfect premating barriers and considerable overlap in larval habitat use, yet without one outcompeting another over time [8, 45]. To foster food and economic security, the production of rice in Africa has more than doubled in percentage from 1.76% between 1999–2001 to 3.96% between 2002–2013 [46]. Rice exports from the region have also increased in recent years with more landmass dedicated to irrigated rice farming [47]. Advancement in agricultural technology and increased funding and support to the farmer have established rice cultivation as a year-round activity alien to its prior seasonal cycle [47]. These activities create new ecological larval niches for *An. coluzzii* and thus new opportunities for their specialisation and expansion into novel habitats as evidenced by the high density of the species in regions with intensive farming [8, 27, 43, 45, 46, 48].

Of direct translational impact, the results of this study can inform modifications in *An. gambiae* (s.l.) rearing protocols to include mineral water instead of the commonly used deionised water. Earlier studies have demonstrated that the use of mineral water in rearing *An. gambiae* (s.s.) and *An. coluzzii* significantly improved larval survival and phenotypic quality of emerged adults and led to shorter development time [42, 49]. Ongoing efforts toward mass release mosquitoes modified by gene drive technology, SIT (sterile insect techniques) implementation, and other vector control strategies that rely on large-scale production of mosquitoes could benefit from the introduction of mineral water in mass rearing protocols to improve mosquito yield and adult phenotypic quality.

Additionally, the results from this study provide evidence that microcosms can be a useful model system for further mosquito ecological speciation studies such as testing the effect of larval predation on divergent ecological adaptation or ecotoxicological aspects such as bio-larvicide implementation in rice fields. The microcosm model provides an experimental arena with simplified miniature ecosystems useful for predicting phenotypic responses under controlled conditions [50]. It allows for multiple replications and varying experimental conditions to establish a cause-effect relationship between variables and the test organism to provide valuable information on the ecology of *An. gambiae* (s.l.) populations inhabiting rice field ecosystems in the laboratory [51]. Future studies could aim at further adjusting the environmental conditions in the rice paddy and rain puddle habitat to achieve more symmetrical fitness reaction norms across microcosms. Aside from differences in levels of water mineralisation and ammonia levels and higher water depth, the rice paddy microcosms also featured a layer of darker sand on the bottom of the tank and artificial rice plants. Therefore, the possibility that these also contributed to the observed G × E needs to be explored.

One of the crucial components of the study pertained to choosing strains of *An. gambiae* and *An. coluzzii* that were old enough to avoid the possible confounding factor of recent adaptation to environmental contaminants [27, 52]. Older mosquito lines pre-date the M and S diagnostic [53] and hence could be a mixture of both species. Consequently, we chose the Mopti 2003 strain, because it is the oldest of the pure *An. coluzzii* strains, and it was colonised from a population originating from a rural setting with no evidence of significant habitat contamination. The Kisumu strain of *An. gambiae* was also colonised at a time when contaminations from urbanisation were still low. This strain is also well known because it lacks any pesticide resistance mechanisms [54]. It is noteworthy that metabolic insecticide resistance and ammonia detoxification pathways in mosquitoes have been described as distinct [55–57]. Even in the unlikely event of an unknown overlap between insecticide and ammonia detoxification pathways, the possibility of metabolic resistance in the *An. coluzzii* strain used in this study is highly unlikely. P450 genes conferring metabolic resistance to pyrethroids were not detected in *An. coluzzii* populations sampled in Mali prior to the recent selective introgression of these genes from *An. gambiae* (s.s.) following the intensification of pyrethroid-impregnated bednet distributions that started in 2005 [58–60]. Therefore, recent introgression, increased chemical control, and exposure to urban contaminants all combine to complicate the use of younger strains for such studies.

## Conclusions

This study provides supporting evidence for larval ecological divergence amongst the sibling species that resulted in ecological speciation and the sympatric occurrence of the sibling species without out-competing each other. The microcosm model developed in this study highlights their advantage compared to over-simplified experimental set-ups. These could be of further use to investigate other complex divergent adaptive traits in the sibling species such as oviposition site preference, larval responses to aquatic predators, and other traits. Ecological speciation in these sibling species has strong implications for existing vector control measures and the epidemiology of malaria. Larvicidal and adulticidal based vector control methods clearly need to be adjusted to account for the niche expansion, year-round presence, and increasing distribution of *An. coluzzii* resulting from its higher tolerance for eutrophic habitats. The results from this study will hopefully add to the literature that will serve to inform policy decisions to mitigate the fast expansion of *An. coluzzii* larval breeding habitats in the form of irrigated rice cultivation.

## Supplementary information

**Additional file 1: Figure S1.** Experimental set-up for the effect of ammonia concentrations on *An. coluzzii* and *An. gambiae* (s.s.) development (Experiment 1).

**Additional file 2: Figure S2.** Experimental design for the contrasted microcosms experiment (Experiment 2).

**Additional file 3: Figure S3.** Effect of increasing ammonia concentrations on larval survival (Experiment 1). Whiskers represent 95% confidence intervals.

**Additional file 4: Figure S4:** Effect of ammonia and feed regimes on pupal mortality (Experiment 1). The percentage pupal mortality for *An. coluzzii* (**a**) and *An. gambiae* (**b**) for solution (light blue) and powder feed (dark blue). Whiskers represent 95% confidence intervals.

**Additional file 5: Figure S5:** Effect of ammonia on adult emergence (Experiment 1). **a** Overall for both species. **b** *An. coluzzii* feed regimes. **c** *An. gambiae* (s.s.) feed regimes. Bar plots (solution, light blue); (powder, dark blue), show the percentage adult emergence across 7 ammonia concentrations. Whiskers represent 95% confidence intervals.

**Additional file 6: Table S1.** Effect of ammonia, water types and feed regimes on body size (wing length) and day of emergence (Experiment 1).

**Additional file 7: Table S2.** Adult emergence of *An. gambiae* (s.s.) and *An. coluzzii* across two larval microcosms (Experiment 2).

**Additional file 8: Table S3.** Mean wing length and development time of *An. gambiae* (s.s.) and *An. coluzzii* in divergent larval microcosms (Experiment 2).

## Data Availability

All datasets generated and/or analysed during this study are included in this published article and its Additional files.
