# Supplementary figures and images for: Laboratory and microcosm experiments reveal contrasted adaptive responses to ammonia and water mineralisation in aquatic stages of the sibling species Anopheles gambiae (sensu stricto) and Anopheles coluzzii
Source: Parasit Vectors. 2021 Jan 6;14:17. doi: 10.1186/s13071-020-04483-7 (PMC7789177; doi:10.1186/s13071-020-04483-7)

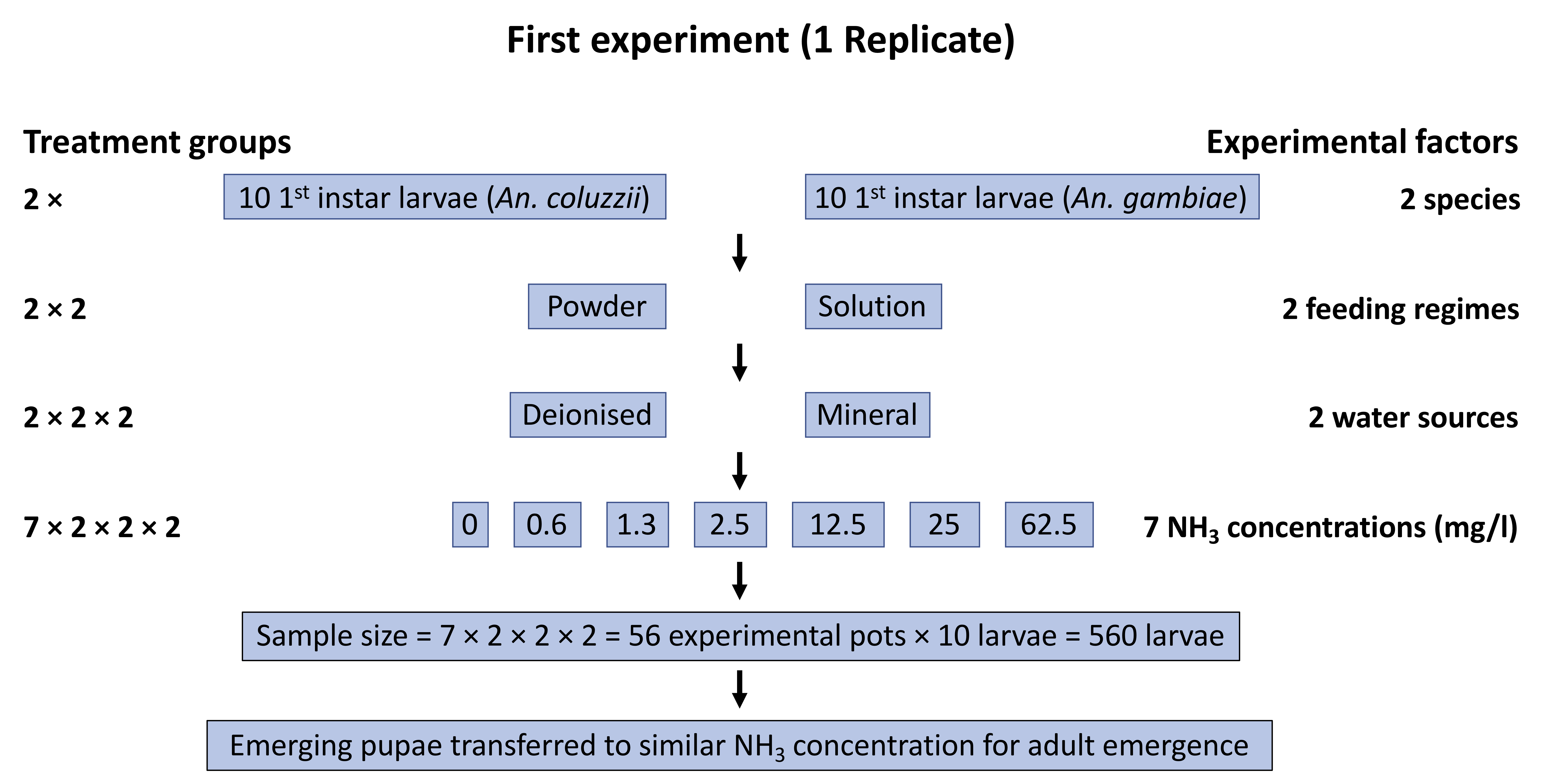

Supplement: Supplementary file 1 — Additional file 1: Figure S1. Experimental set-up for the effect of ammonia concentrations on An. coluzzii and An. gambiae (s.s.) development (Experiment 1). [file 13071_2020_4483_MOESM1_ESM.png]

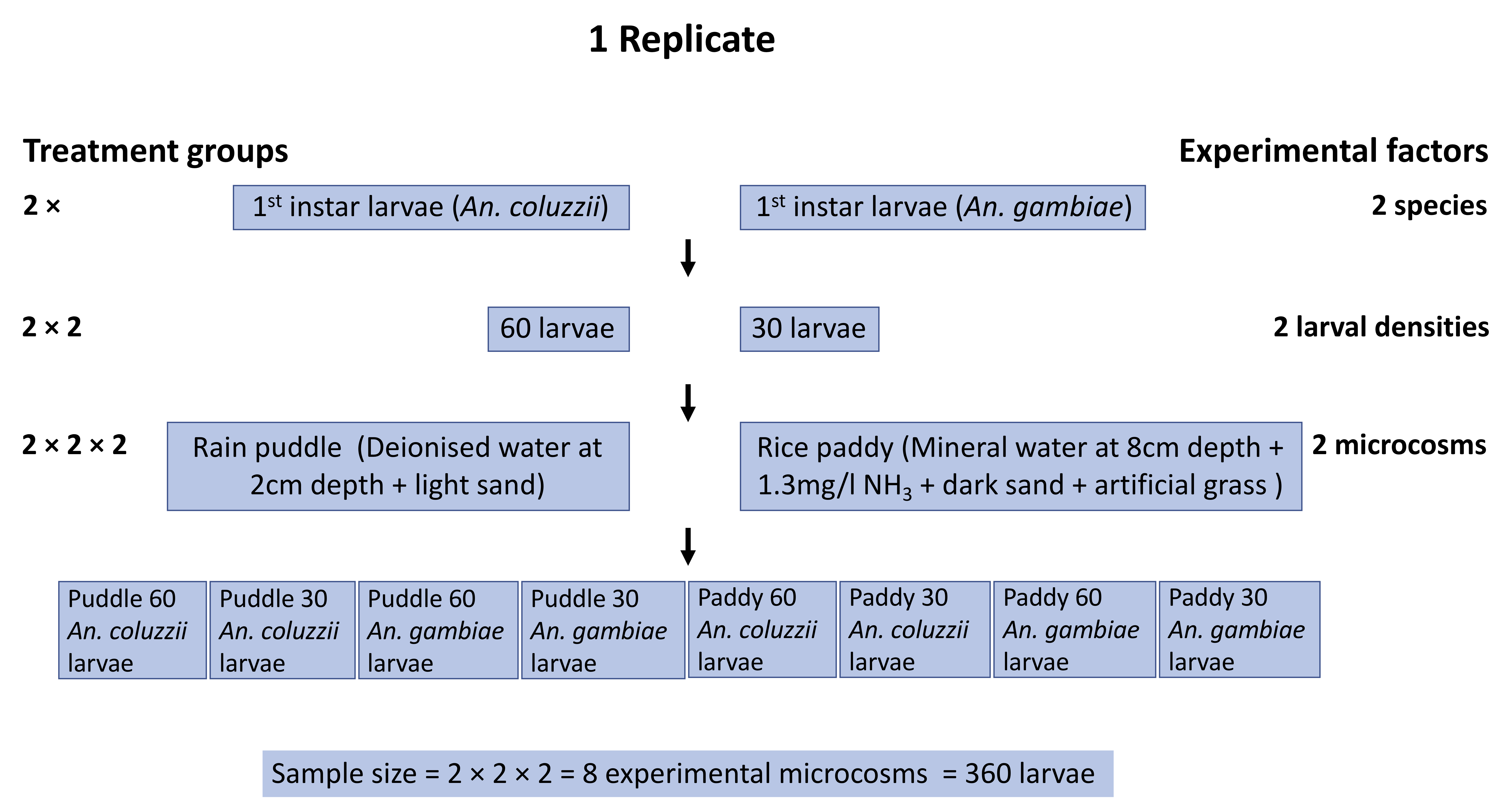

Supplement: Supplementary file 2 — Additional file 2: Figure S2. Experimental design for the contrasted microcosms experiment (Experiment 2). [file 13071_2020_4483_MOESM2_ESM.png]

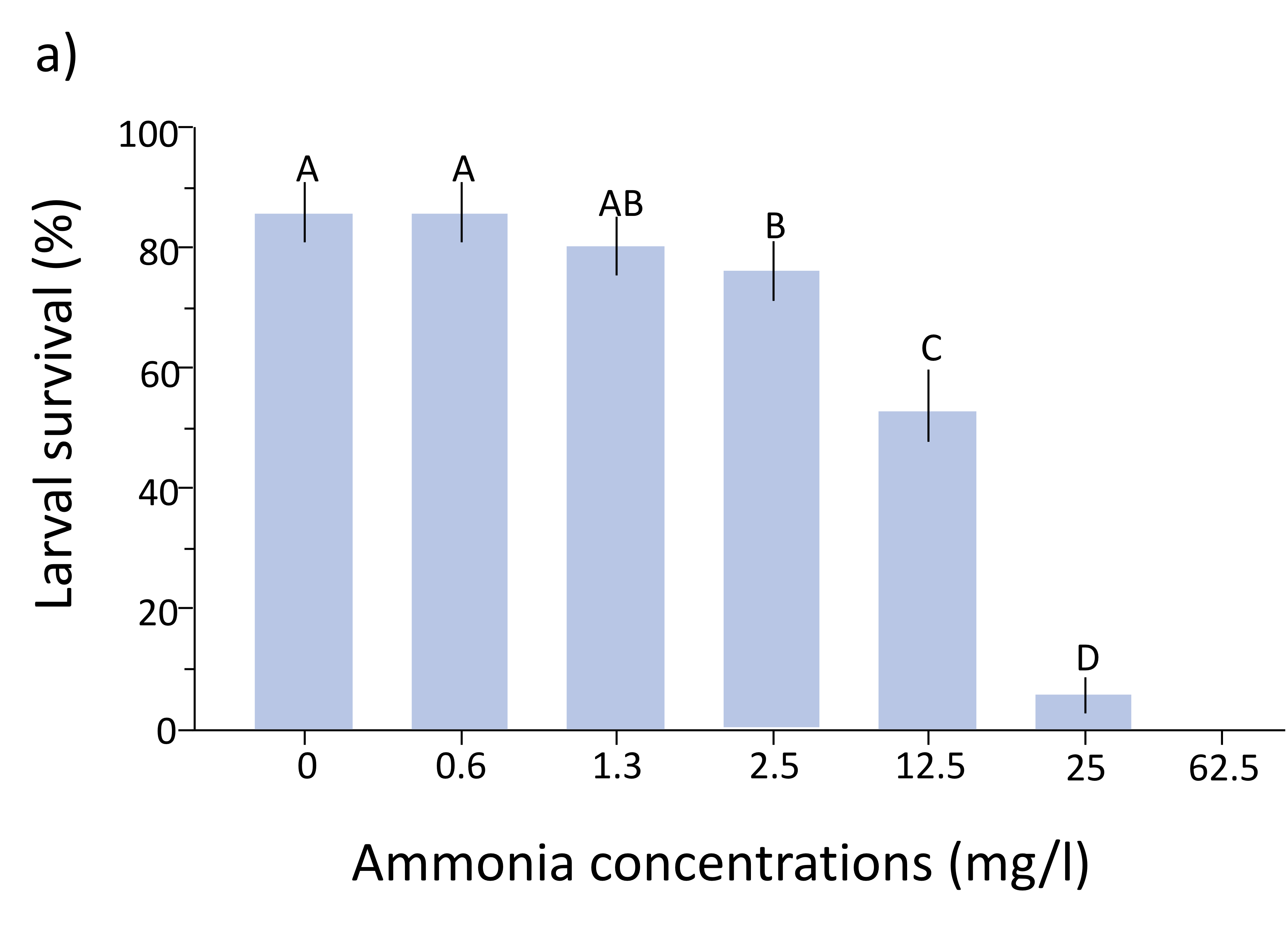

Supplement: Supplementary file 3 — Additional file 3: Figure S3. Effect of increasing ammonia concentrations on larval survival (Experiment 1). Whiskers represent 95% confidence intervals. [file 13071_2020_4483_MOESM3_ESM.png]

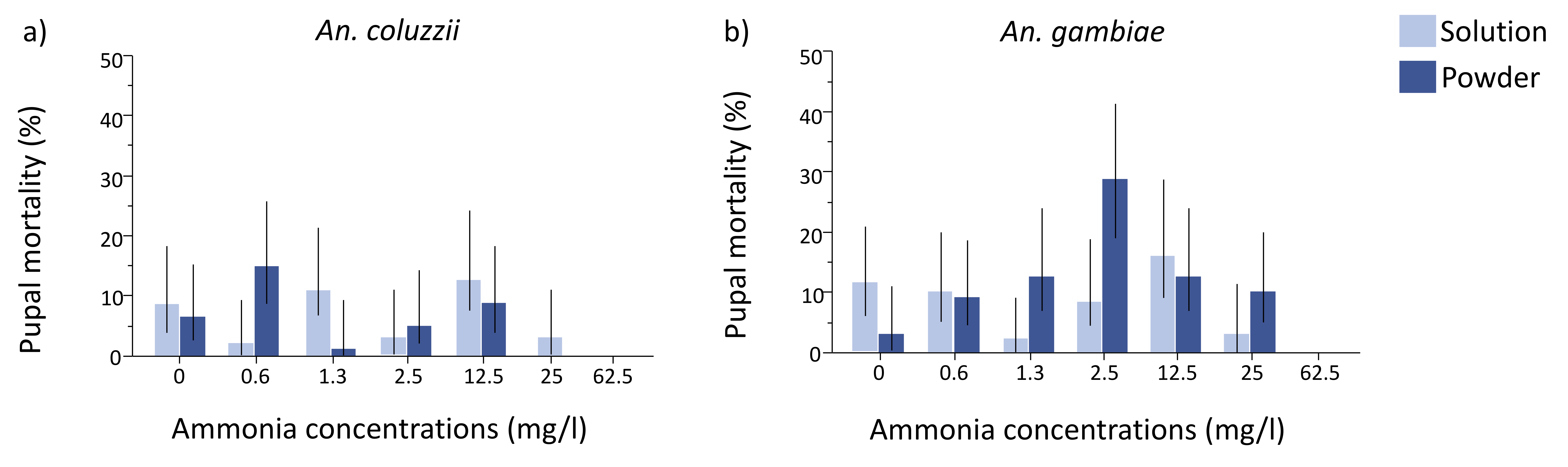

Supplement: Supplementary file 4 — Additional file 4: Figure S4: Effect of ammonia and feed regimes on pupal mortality (Experiment 1). The percentage pupal mortality for An. coluzzii (a) and An. gambiae (b) for solution (light blue) and powder feed (dark blue). Whiskers represent 95% confidence intervals. [file 13071_2020_4483_MOESM4_ESM.png]

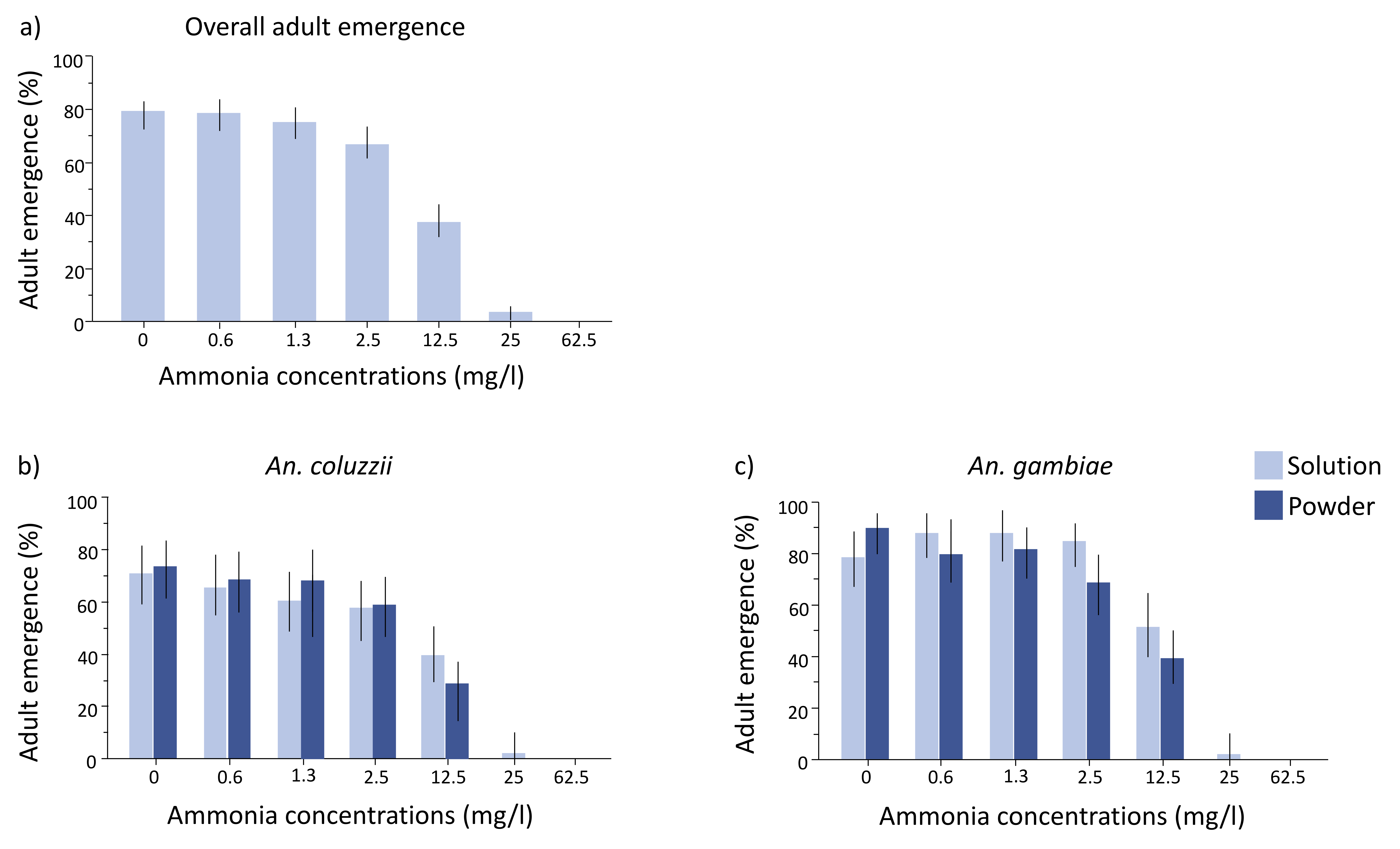

Supplement: Supplementary file 5 — Additional file 5: Figure S5: Effect of ammonia on adult emergence (Experiment 1). a Overall for both species. b An. coluzzii feed regimes. c An. gambiae (s.s.) feed regimes. Bar plots (solution, light blue); (powder, dark blue), show the percentage adult emergence across 7 ammonia concentrations. Whiskers represent 95% confidence intervals. [file 13071_2020_4483_MOESM5_ESM.png]
