# Supplementary material for: Laboratory and microcosm experiments reveal contrasted adaptive responses to ammonia and water mineralisation in aquatic stages of the sibling species Anopheles gambiae (sensu stricto) and Anopheles coluzzii
Source: Parasit Vectors. 2021 Jan 6;14:17. doi: 10.1186/s13071-020-04483-7 (PMC7789177; doi:10.1186/s13071-020-04483-7)
Supplement: Supplementary file 6 — Additional file 6: Table S1. Effect of ammonia, water types and feed regimes on body size (wing length) and day of emergence (Experiment 1). [file 13071_2020_4483_MOESM6_ESM.pdf]

Species	Water type	Feed regime	Ammonia (mg/l)	Mean wing length (mm)	Days till emergence
An. coluzzii	Deionised	Solution	0	3.00 (2.86–3.13) *20*	9.85 (9.41–10.29) 20
			0.6	2.95 (2.86-3.04) *22*	9.77 (9.44-10.11) *22*
			1.3	2.81 (2.68-2.93) *17*	10.24 (9.67-10.80) *17*
			2.5	2.81 (2.66-2.95) *16*	9.75 (9.25-10.25) *16*
			12.5	2.9 (2.24-3.56) *3*	11.33 (9.89-12.77) *3*
			25	0	0
			62.5	0	0
		Powder	0	3.00 (2.87–3.11) *22*	9.82 (9.52–10.11) *22*
			0.6	2.85 (2.74-2.97) *17*	9.76 (9.38-10.15) *17*
			1.3	2.88 (2.77-2.98) *24*	10.42 (10.14-10.69) *24*
			2.5	2.76 (2.58-2.94) *14*	10.29 (9.81-10.76) *14*
			12.5	0	0
			25	0	0
			62.5	0	0
	Mineral	Solution	0	3.07 (2.98–3.16) *22*	9.64 (9.31–9.96) *22*
			0.6	2.98 (2.89-3.07) *23*	9.74 (9.44-10.04) *23*
			1.3	2.92 (2.80-3.04) *19*	9.37 (9.13-9.61) *19*
			2.5	3.04 (2.94-3.13) *19*	9.68 (9.36-10.01) *19*
			12.5	2.97 (2.88-3.07) *21*	9.67 (9.40-9.93) *21*
			25	2.90 (2.90-2.90) *2*	10.5 (-8.56-29.56) *2*
			62.5	0	0
		Powder	0	3.03 (2.93–3.13) *22*	9.86 (9.40–10.32) *22*
			0.6	3.04 (2.95-3.14) *23*	9.43 (9.14-9.72) *23*
			1.3	3.01 (2.86-3.16) *17*	9.06 (8.77-9.34) *17*
			2.5	2.98 (2.92-3.04) *20*	9.50 (9.22-9.78) *20*
			12.5	2.93 (2.75-3.10) *16*	9.63 (9.30-9.95) *16*
			25	0	0
			62.5	0	0
An. gambiae	Deionised	Solution	0	2.97 (2.86–3.08) 24	10.38 (10.10–10.65) 24
			0.6	3.02 (2.95-3.08) 26	10.15 (9.91-10.40) 26
			1.3	2.97 (2.89-3.04) 27	10.41 (10.16-10.66) 27
			2.5	2.95 (2.83-3.07) 21	10.29 (10.08-10.50) 21
			12.5	2.77 (2.55-2.98) 6	12.67 (11.23-14.10) 6
			25	0	0
			62.5	0	0
		Powder	0	3.07 (2.96–3.18) *28*	10.36 (10.00–10.71) *28*
			0.6	2.99 (2.91-3.08) *22*	10.23 (9.96-10.50) *22*
			1.3	2.92 (2.84-2.99) *20*	10.35 (10.12-10.58) *20*
			2.5	2.93 (2.81-3.05) *16*	10.75 (10.51-10.99) *16*
			12.5	0	0
			25	0	0
			62.5	0	0
	Mineral	Solution	0	3.06 (2.98 –3.14) *23*	9.43 (9.22–9.65) *23*
			0.6	3.05 (2.98-3.13) *26*	9.85 (9.60-10.09) *26*
			1.3	2.98 (2.90-3.06) *25*	10.08 (9.88-10.28) *25*
			2.5	2.82 (2.72-2.93) 30	9.60 (9.39-9.81) 30
			12.5	2.88 (2.76-2.99) *25*	10.76 (10.51-11.01) *25*
			25	2.6 (-2.48-7.68) *2*	11.50 (5.15-17.85) *2*
			62.5	0	0
		Powder	0	3.08 (3.00–3.17) *26*	9.50 (9.29 – 9.71) *26*
			0.6	3.06 (2.98-3.15) *26*	9.54 (9.28-9.80) *26*

Species	Water type	Feed regime	Ammonia (mg/l)	Mean wing length (mm)	Days till emergence
			1.3	2.95 (2.84-3.05) *29*	9.79 (9.61-9.98) *29*
			2.5	3.02 (2.90-3.14) *25*	9.48 (9.27-9.69) *25*
			12.5	2.81 (2.67-2.94) *23*	10.35 (10.14-10.56) *23*
			25	0	0
			62.5	0	0

Notes: Ninety-five percent confidence intervals are in parentheses and the samples sizes, the number of surviving individuals out of an initial number of 30 larvae are italicized
