# Supplementary material for: Laboratory and microcosm experiments reveal contrasted adaptive responses to ammonia and water mineralisation in aquatic stages of the sibling species Anopheles gambiae (sensu stricto) and Anopheles coluzzii
Source: Parasit Vectors. 2021 Jan 6;14:17. doi: 10.1186/s13071-020-04483-7 (PMC7789177; doi:10.1186/s13071-020-04483-7)
Supplement: Supplementary file 7 — Additional file 7: Table S2. Adult emergence of An. gambiae (s.s.) and An. coluzzii across two larval microcosms (Experiment 2). [file 13071_2020_4483_MOESM7_ESM.pdf]

Species	Microcosm	Larval density	% Adult emergence
An. coluzzii	Rice paddy	30	18 (11-30) *60*
		60	16 (10-23) *120*
	Rain puddle	30	32 (21- 44) *60*
		60	42 (33-51) *120*
An. gambiae s.s	Rice paddy	30	2 (0-9) *60*
		60	13 (9-21) *120*
	Rain puddle	30	76 (64-86) *60*
		60	63 (54-71)*120*

Ninety-five percent confidence intervals are in brackets and sample sizes are italicized. Adult emergence was calculated as emerged adults out of the initial sample size.
