# Supplementary material for: Laboratory and microcosm experiments reveal contrasted adaptive responses to ammonia and water mineralisation in aquatic stages of the sibling species Anopheles gambiae (sensu stricto) and Anopheles coluzzii
Source: Parasit Vectors. 2021 Jan 6;14:17. doi: 10.1186/s13071-020-04483-7 (PMC7789177; doi:10.1186/s13071-020-04483-7)
Supplement: Supplementary file 8 — Additional file 8: Table S3. Mean wing length and development time of An. gambiae (s.s.) and An. coluzzii in divergent larval microcosms (Experiment 2). [file 13071_2020_4483_MOESM8_ESM.pdf]

Species	Microcosm	Larval density	Mean wing length (mm)	Days till emergence
An. coluzzii	Rice paddy	30	3.00 (2.81–3.18) 11	12.55 (11.68-13.41) 11
		60	2.82 (2.75–2.89) 19	12.84 (12.33-13.36) 19
	Rain puddle	30	3.22 (3.12–3.31) 19	10.95 (10.61-11.29) 19
		60	3.09 (3.04–3.13) 50	11.24 (11.02-11.46) 50
An. gambiae	Rice paddy	30	2.68 (0–0) 1	12 (0-0) 1
		60	2.88 (2.81–2.94) 16	12.94 (12.53-13.35) 16
	Rain puddle	30	3.04 (2.99 –3.08) 46	10.76 (10.55-10.97) 46
		60	3.07 (3.04–3.09) 75	10.99 (10.77-11.20) 75

Notes: Ninety-five percent confidence intervals are in parentheses and the samples sizes, the number of surviving individuals out of an initial number of 60 or 120 larvae are italicized.
